# peakPantheR, an R package for large-scale targeted extraction and integration of annotated metabolic features in LC–MS profiling datasets

**DOI:** 10.1093/bioinformatics/btab433

**Published:** 2021-06-14

**Authors:** Arnaud M Wolfer, Gonçalo D S Correia, Caroline J Sands, Stephane Camuzeaux, Ada H Y Yuen, Elena Chekmeneva, Zoltán Takáts, Jake T M Pearce, Matthew R Lewis

**Affiliations:** National Phenome Centre, Department of Metabolism, Digestion and Reproduction, Imperial College London, Hammersmith Campus, IRDB Building, London W12 0NN, UK; Roche Pharma Research and Early Development, pRED Informatics, Roche Innovation Center Basel, F. Hoffmann-La Roche Ltd, Basel CH 4070, Switzerland; National Phenome Centre, Department of Metabolism, Digestion and Reproduction, Imperial College London, Hammersmith Campus, IRDB Building, London W12 0NN, UK; Section of Bioanalytical Chemistry, Department of Metabolism, Digestion and Reproduction, Imperial College London, South Kensington Campus, London SW7 2AZ, UK; National Phenome Centre, Department of Metabolism, Digestion and Reproduction, Imperial College London, Hammersmith Campus, IRDB Building, London W12 0NN, UK; Section of Bioanalytical Chemistry, Department of Metabolism, Digestion and Reproduction, Imperial College London, South Kensington Campus, London SW7 2AZ, UK; National Phenome Centre, Department of Metabolism, Digestion and Reproduction, Imperial College London, Hammersmith Campus, IRDB Building, London W12 0NN, UK; Section of Bioanalytical Chemistry, Department of Metabolism, Digestion and Reproduction, Imperial College London, South Kensington Campus, London SW7 2AZ, UK; National Phenome Centre, Department of Metabolism, Digestion and Reproduction, Imperial College London, Hammersmith Campus, IRDB Building, London W12 0NN, UK; Section of Bioanalytical Chemistry, Department of Metabolism, Digestion and Reproduction, Imperial College London, South Kensington Campus, London SW7 2AZ, UK; National Phenome Centre, Department of Metabolism, Digestion and Reproduction, Imperial College London, Hammersmith Campus, IRDB Building, London W12 0NN, UK; Section of Bioanalytical Chemistry, Department of Metabolism, Digestion and Reproduction, Imperial College London, South Kensington Campus, London SW7 2AZ, UK; National Phenome Centre, Department of Metabolism, Digestion and Reproduction, Imperial College London, Hammersmith Campus, IRDB Building, London W12 0NN, UK; Section of Bioanalytical Chemistry, Department of Metabolism, Digestion and Reproduction, Imperial College London, South Kensington Campus, London SW7 2AZ, UK; National Phenome Centre, Department of Metabolism, Digestion and Reproduction, Imperial College London, Hammersmith Campus, IRDB Building, London W12 0NN, UK; National Phenome Centre, Department of Metabolism, Digestion and Reproduction, Imperial College London, Hammersmith Campus, IRDB Building, London W12 0NN, UK; Section of Bioanalytical Chemistry, Department of Metabolism, Digestion and Reproduction, Imperial College London, South Kensington Campus, London SW7 2AZ, UK

## Abstract

**Summary:**

Untargeted liquid chromatography–mass spectrometry (LC–MS) profiling assays are capable of measuring thousands of chemical compounds in a single sample, but unreliable feature extraction and metabolite identification remain considerable barriers to their interpretation and usefulness. peakPantheR (*Peak Picking and ANnoTation of High-resolution Experiments in R*) is an R package for the targeted extraction and integration of annotated features from LC–MS profiling experiments. It takes advantage of chromatographic and spectral databases and prior information of sample matrix composition to generate annotated and interpretable metabolic phenotypic datasets and power workflows for real-time data quality assessment.

**Availability and implementation:**

peakPantheR is available via Bioconductor (https://bioconductor.org/packages/peakPantheR/). Documentation and worked examples are available at https://phenomecentre.github.io/peakPantheR.github.io/ and https://github.com/phenomecentre/metabotyping-dementia-urine.

**Supplementary information:**

Supplementary data are available at *Bioinformatics* online.

## 1 Introduction

Liquid chromatography–mass spectrometry (LC–MS) is a key analytical platform in modern metabolic phenotyping workflows, owning to its sensitivity and broad chemical coverage. A state-of-the-art LC–MS metabolic profiling assay is capable of detecting >10 000 ion species in a single sample (Ivanisevic *et al*., 2013; Lewis *et al*., 2016; Naser *et al*., 2018). This information is commonly extracted with untargeted peak picking algorithms. These algorithms attempt to extract as many peaks as possible from each sample, and account for sample-to-sample analytical variation by establishing correspondences between similar signals across samples, combining peaks into groups known as features. The end product is a data matrix of samples and features which can be filtered to reduce variable inflation due to false positives at the peak detection stage and remove poor quality measurements. Chemical assignment is then performed by matching feature’s retention time and *m*/*z* values to spectral databases. An alternative and more direct approach is to tackle LC–MS data pre-processing as a targeted feature extraction problem, prioritizing ion species peaks known to be well captured by the analytical methodology. Advances in the characterization of metabolomes (Wishart *et al*., 2018) and LC–MS assays (Tada *et al*., 2019), including the improved quality of spectral and chromatographic databases, make such an approach more tractable, even for application to complex sample matrices. However, existing software for targeted feature extraction is more tailored to the integration of a limited number of features in targeted LC–MS triple quadrupole experiments, with interfaces and visualization designed for examination of each individual signal in each sample at the cost of extensive manual intervention. While appropriate for supporting targeted bioanalysis workflows, they are impractically applied to high-resolution global profiling data. For this reason, an unmet need exists for automated, scalable and high-throughput targeted annotation and integration software that is suited to the extraction of hundreds of features in large LC–MS profiling experiments.

**Fig. 1.**
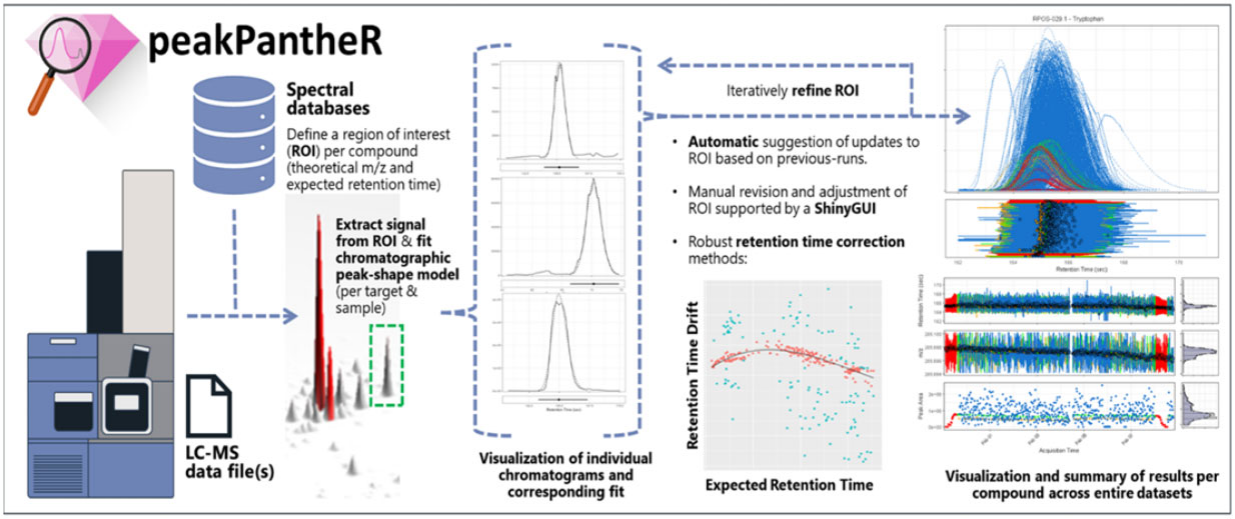
Overview of the peakPantheR package functionality and example outputs

To address this, we have developed peakPantheR (*Peak Picking and ANnoTation of High-resolution Experiments in R*), an open-source R package for targeted extraction and integration of annotated chemical compounds from untargeted LC–MS profiling datasets. peakPantheR leverages prior knowledge of LC–MS performance characteristics and provides users with both an automated data extraction solution and direct interface for manual refinement where necessary (see Figure 1).

## 2 The peakPantheR package

### 2.1 Implementation

peakPantheR is an open-source R (v4.0.0 or above) package and is available via Bioconductor (https://bioconductor.org/packages/peakPantheR/). The main functionality is command-line based, but a shiny graphical user interface (GUI) is provided to assist users in visualizing and iteratively refining the integration region boundary and parameters. Emphasis was placed on providing visualization options and diagnostic metrics adequate for inspection of results at dataset level, to facilitate robust high-throughput analysis. Tutorial vignettes exemplifying the main functions are available via Bioconductor. An example application to a cohort of 600 human urine biofluid samples profiled by three complementary LC–MS assays can be found in https://github.com/phenomecentre/metabotyping-dementia-urine. In this example, 315 annotated ion species are extracted using peakPantheR from three LC–MS assays described by Lewis *et al.* (2016). Detailed instructions manual is also available in the Supplementary File S1.

### 2.2 Features

peakPantheR workflows are structured around the *peakPantheRAnnotation* object, which represents the outcome of a targeted search and integration of signals in a series of pre-specified regions of interest (ROI). The required inputs for peakPantheR are the raw MS data files in mzML or any format supported by mzR (Chambers *et al*., 2012) and a comma-separated file defining the retention time and *m*/*z* boundaries for the ROI to integrate. Although designed for centroided data, profile/continuum data are supported. Functionality to run peakPantheR in parallel across multiple MS files simultaneously is provided via batch commands.

### 2.3 Chromatographic peak models and quality metrics

The peakPantheR integration model works by fitting a chromatographic line-shape model to the extracted ion chromatograms (EIC) from each ROI. Two line shapes are supported, a skewed Gaussian and an exponential modified Gaussian model. These are specifically tailored for chromatographic signals and can recreate asymmetry and tailing/fronting. If a peak model can be fitted acceptably to the EIC, the line shape is used to obtain the peak integral and other characteristics (i.e. peak width and peak asymmetry), otherwise a fallback integration of the EIC data points is performed, to handle extreme deviations in peak shape. peakPantheR is intended to be applied iteratively to a series of features/samples; to improve the reliability of the integration across the entirety of a dataset, the software automatically suggests refinements of ROI based on dataset-wide consensus estimated from a previous run’s results. Detailed information about the line-shape models, algorithms and metrics estimated can be found in the Supplementary materials.

### 2.4 Retention time adjustment

Retention time values are empirically derived, and therefore systematic deviations from data-based values are expected. Functionality for retention time re-calibration based on expected retention times for calibrants (either spiked internal standards or endogenous compounds) is implemented, including a robust RANSAC (Fischler and Bolles, 1981) method for correction based on endogenous compounds.

### 2.5 Shiny GUI

**Fig. 2.**
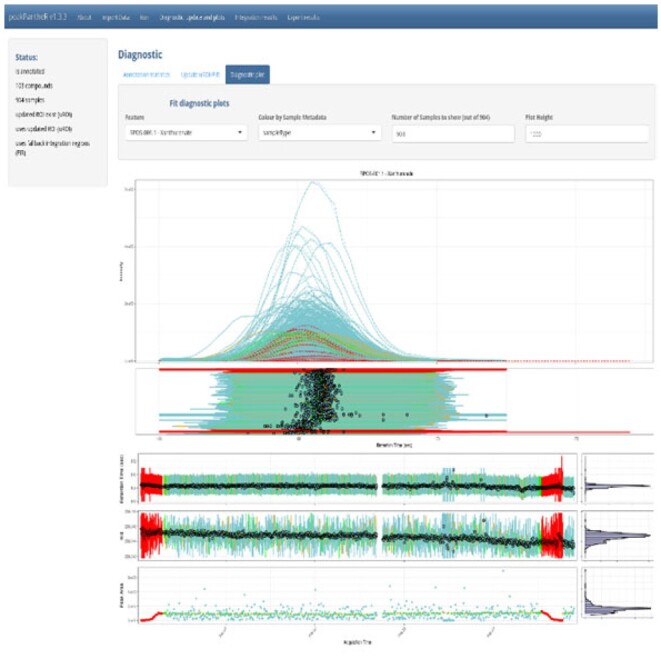
peakPantheR’s shiny graphical user interface

A shiny GUI is available to review peakPantheR’s results (Figure 2). The EIC and the corresponding line-shape fits are displayed in interactive plots, with action buttons and forms so the user can review and adjust the ROI boundaries more easily and re-trigger the integration procedure.

## 3 Concluding remarks

peakPantheR is a general purpose, automated and scalable targeted feature extraction software capable of producing high-fidelity datasets from global profiling LC–MS data. We anticipate it to be a valuable addition to the existing LC–MS data pre-processing toolkit as a key component of targeted integration workflows which take advantage of established chromatographic databases to obtain annotated, interpretable, and ultimately, actionable metabolic phenotypic datasets.

## Funding

This work was supported by the Medical Research Council (MRC) and National Institute for Health Research (NIHR) [grant number MC_PC_12025] and the MRC UK Consortium for MetAbolic Phenotyping (MAP/UK) [grant number MR/S010483/1]. Infrastructure support was provided by the NIHR Imperial Biomedical Research Centre (BRC).

*Conflict of Interest*: none declared.

## Supplementary Material

btab433_Supplementary_Data
