# Supplementary material for: peakPantheR, an R package for large-scale targeted extraction and integration of annotated metabolic features in LC–MS profiling datasets
Source: Bioinformatics. 2021 Jun 14;37(24):4886–8. doi: 10.1093/bioinformatics/btab433 (PMC8665750; doi:10.1093/bioinformatics/btab433)
Supplement: btab433_Supplementary_Data [file btab433_supplementary_data.zip › ppR_Supplementary_Methods_Revision_09042021.docx]

**peakPantheR, an R package for large-scale targeted annotation and integration of metabolic features in LC-MS profiling datasets**

**Supplementary information**

# Package structure and implementation

The *peakPantheR* software package is implemented in the R language^1^, adhering to the S4 object-oriented programming convention for R OOP^2^⁠. Users can use *peakPantheR* on its own or as part of more complex workflows by running the software either in the command line or from a Shiny^3^⁠ graphical user interface (GUI).

The package can be downloaded from Bioconductor using the BiocManager^4^⁠ package (https://bioconductor.org/packages/peakPantheR/):

if (!requireNamespace("BiocManager", quietly = TRUE))

install.packages("BiocManager")

BiocManager::install("peakPantheR")

The development version is available on *github* (<https://github.com/phenomecentre/peakPantheR>) and can be installed directly using the *devtools*^5^*⁠* package or also via BiocManager:

devtools::install_github(“phenomecentre/peakPantheR”, ref=”develop”)

BiocManager::install("phenomecentre/peakPantheR", ref=”develop”)

A getting started tutorial vignette describing the main inputs and data formats are available at <https://bioconductor.org/packages/release/bioc/vignettes/peakPantheR/inst/doc/getting-started.html>, and extra documentation is also available at <https://phenomecentre.github.io/peakPantheR.github.io>.

*peakPantheR* was designed with two main use cases in mind, annotation and integration of features from a single file in real time (immediately after LC-MS data acquisition), and parallel annotation of an entire dataset comprised of multiple LC-MS data files. These are covered and exemplified in the tutorial vignettes, available in the Bioconductor package summary page <https://bioconductor.org/packages/peakPantheR/>:

- **Getting Started with the peakPantheR package:** Describes the basic functionality and how to generate the required tutorial input data.
- **Real Time Annotation:** Example application of peakPantheR for the annotation of multiple features from a single file.
- **Parallel Annotation:** Example application of peakPantheR to extract multiple features from a series of files in parallel.

Besides the vignettes, an example application of peakPantheR to integrate a series of compounds from a human urine biofluid dataset is available via GitHub <https://github.com/phenomecentre/metabotyping-dementia-urine>. This dataset is comprised of urine biofluid samples collected as part of the AddNeuroMed^6^ cohort study, which follows patients longitudinally with the aim of identifying biomarkers of neurocognitive decline and Alzheimer’s disease. A total of 650 baseline spot urine samples (first sample collected after recruitment to the study) were profiled by three LC-MS assays (Reversed-phase positive mode, Reversed-phase negative mode and HILIC-positive mode)^7^. Information about the experimental methods for this dataset can be found below in Section 7 – “*Experimental details for the dementia cohort dataset and LC-MS metabolite identification*” and in the Lewis et al^7^ publication. The raw data and derived mzML files from the three LC-MS assays can be found in the MetaboLights Study MTBLS719 (<https://www.ebi.ac.uk/metabolights/MTBLS719/>). The github repository contains a set of metabolite annotations, already in the required input format, and example scripts to apply peakPantheR to extract these compounds from the data files, as well as examples of the expected outputs. An additional tutorial for the quality control and preprocessing of the peakPantheR outputs using the nPYc-toolbox^8^ is available at <https://github.com/phenomecentre/nPYc-toolbox-tutorials>.

The main package dependencies of *peakPantheR* are *mzR*^9^*⁠*, *MSnbase*^10^, *minpack.lm*^11^, *Shiny*^3^*⁠*, *ggplot2*^12^*⁠* and *BiocParallel*^13^*.* *mzR* and *MSnbase* are core packages for LC-MS data parsing, and their use for MS file access ensures stable and up-to-date compatibility with the widest range of vendor specific file formats. All these are available through CRAN and/or Bioconductor (using BiocManager^4^⁠) and will be installed automatically when following the installation instructions above.

# General usage and features

*PeakPantheR* can be used via the command line or with the help of the Shiny GUI. The underlying workflows are the same, as the Shiny GUI solely provides an interactive interface to visualize and modify the parameters and integration results and call the command line functions on demand. In this section, we describe the main functionality and features of peakPantheR with command-line examples. For a description of the Shiny GUI functionality please see the Section *6 – “Graphical user interface”*.


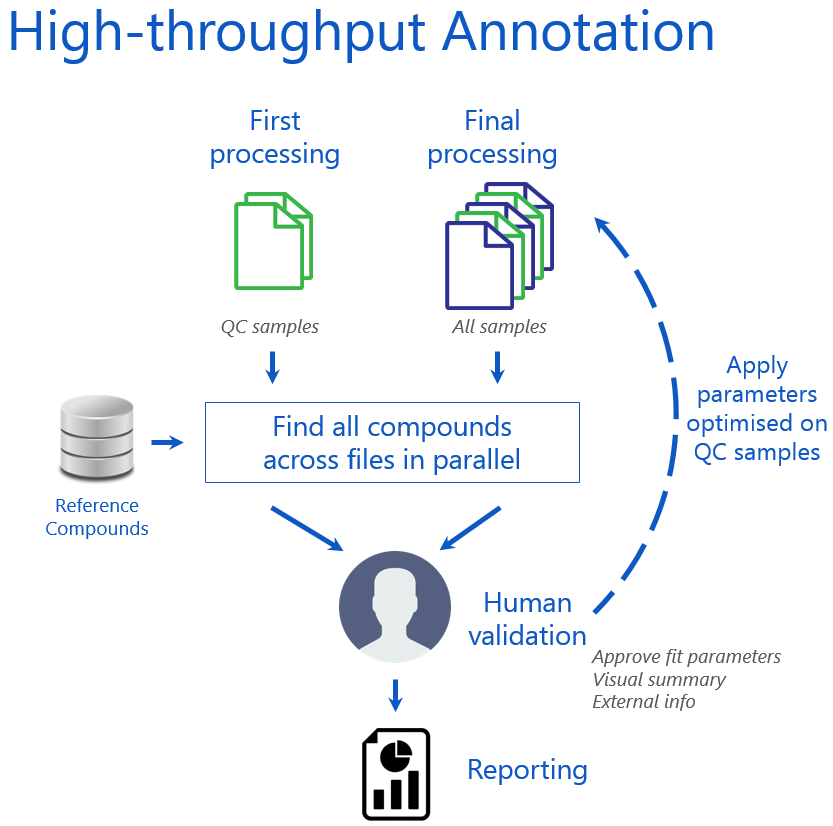


**Figure 1** - For optimal results, the *peakPantheR* workflow relies on iterative peak integration: at first a subset of representative samples spanning the duration of the run (e.g., quality control samples) are processed and features of interest integrated. Once the regions of interest (ROI) have been adapted as necessary using the diagnostic plots (*Figure 4*) as guidance to ensure all features are integrated correctly, the complete dataset can be processed and a final report generated.

In the context of LC-MS profiling and *peakPantheR*, an annotation is the mapping of an identifier, such as a chemical identifier, to a signal or region of interest in the raw data (retention time and mass-to-charge m/z values). The main object in *peakPantheR* is the *peakPantheRAnnotation* class which represents and stores the information and outputs from annotation and integration of LC-MS metabolomic profiling experiments. *PeakPantheR* targeted annotation and integration workflows are provided through the methods of this class. It contains attributes which store spectra and feature metadata, filesystem paths to the raw LC-MS files, information on annotation and integration regions and outputs of the annotation and integration process.

There are three main types of annotation and targeted integration regions in *peakPantheR*:

• Region Of Interest (ROI): original reference RT and m/z windows in which to search for a peak

• updated Region of Interest (uROI): user modified ROI adapted to the current dataset, these override the reference ROI if defined

• Fallback Integration Region (FIR): RT and m/z window to integrate if no peak is found

**Initialisation (*peakPantheRAnnotation*) and required input files:**

The following code block shows the syntax used to initialise a *peakPantheRAnnotation* object:

init_annotation <- peakPantheRAnnotation(spectraPaths = input_spectraPaths,

targetFeatTable = input_targetFeatTable,

spectraMetadata = input_spectraMetadata)

This requires passing a list of raw data files to annotate in *spectraPaths* and a set of annotations (target ROIs) to search and integrate, the *targetFeatTable.* Metadata for each LC-MS spectra can also be passed as a dataframe, the *input_spectraMetadata.* At this stage, the object’s *isAnnotated* attribute is set to FALSE, as the LC-MS files have not been accessed nor any target features extracted and integrated.

*peakPantheR* uses the *MSnbase* package and its OnDiskMSnExp object to interface with the raw data files, therefore *input_spectraPaths* can point to any file format recognized and supported by *MSnbase* and *mzR.* We recommend using the .mzML open format^14^; netCDF, mzXML^15^⁠ or mzData are also supported.

*peakPantheR* takes a csv file with predefined regions of interest (ROI) as the *input_targetFeatTable*. The ROI is the primary set of retention time and *m/z* window values passed to *targetFeatTable* when initialising the *peakPantheRAnnotation* object. It records the minimum annotation information required by the software and the expected reference values for a set of targets*.* An example of the structure required to define a set of ROI is provided in ***Table 1***. Any additional information passed as extra columns (for example, external database identifiers, such as KEGG IDs) will be preserved.

| **cpdID** | **cpdName** | **rtMin** | **Rt** | **rtMax** | **mzMin** | **mz** | **mzMax** |
| --- | --- | --- | --- | --- | --- | --- | --- |
| 1 | Cpd 1 | 3310 | 3344.888 | 3390 | 522.194778 | 522.2 | 522.205222 |
| 2 | Cpd 2 | 3280 | 3385.577 | 3440 | 496.195038 | 496.2 | 496.204962 |

**Table 1** - Example of the ROI definition table and the required column fields. Retention time is defined in seconds (s).

The *peakPantheRParallelAnnotation* function automatically handles the parallel annotation of multiple files and returns a list containing a modified *peakPantheRAnnotation* object with the results and a log of any warnings and errors that occurred during processing. This log stores only errors and exceptions associated with file reading and parsing, not the specific outcomes of the targeted annotation process for each target.

**Targeted annotation and integration (*peakPantheR_parallelAnnotation*):**

The following command starts the *peakPantheR* targeted annotation and integration process over all registered raw LC-MS files:

annotation_result <- peakPantheR_parallelAnnotation(init_annotation, ncores=8, resetWorkers=30, verbose=TRUE)

The *ncores* argument defines how many CPU cores to use. The *resetWorkers* argument sets a limit on how many files each worker processes before restarting the back-end parallel engine. This step is performed to release physical memory when processing a large number of files over time. The *ncores* argument controls the number of cores used in parallel and should be tweaked to find a compromise between using a large number of cores and not exceeding the available physical memory, since reading MS files can be memory intensive. We recommend first running with a smaller number of cores (4 to 6) to evaluate the physical memory usage, and then increasing the *ncores* parameter accordingly. Also, the magnitude of the speed up obtained when doing parallel integration is contingent on the disk I/O speed capacity.


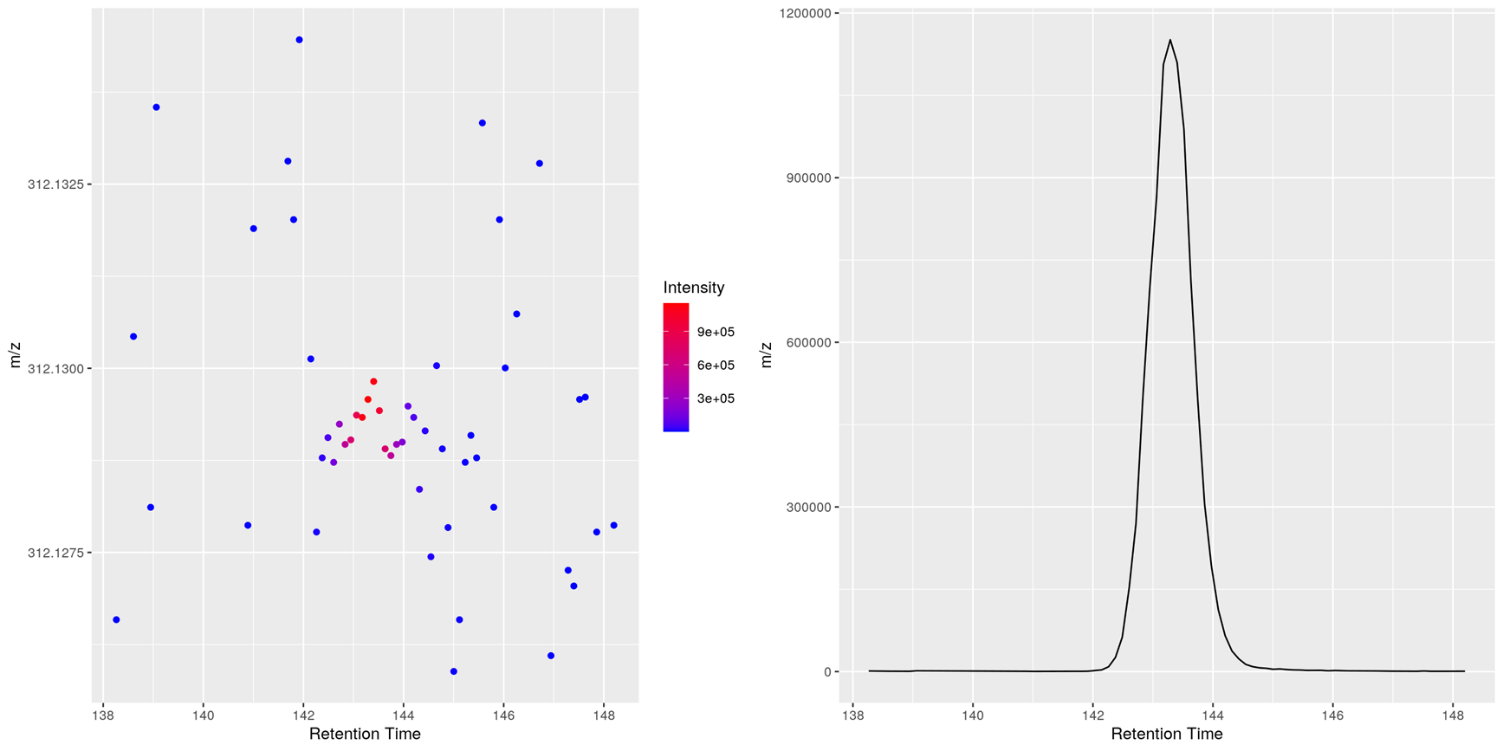


**Figure 2** – Raw LC-MS centroided data extracted from the region of interest (ROI) parameters defined in Table 3. The left plot is a 2D view of the individual data points (intensity indexed by m/z and retention time) present in the ROI. The corresponding extracted ion chromatogram (EIC), shown in the right panel is obtained by summing the intensity from all data points accross the m/z dimension.

For each ROI (for example, as defined in Table 3), the data points contained within the m/z and retention time window are read and aggregated into an extracted ion chromatogram (EIC) (***Figure 2***). An EIC contains only one intensity value per retention time value/scan, obtained by summing all the *m/z* values with the defined range per a scan. At this stage, a simple check is performed to ensure signal is present in the EIC for the region of interest (ROI). If the EIC is not empty (at least one scan contains a non-zero intensity entry), a chromatographic peak is fitted to the data points (***Figure 3***) and used to extract the peak integral and other quantities of interest (see ***Table 2***). Information about the chromatographic peak models, the peak fitting procedure and diagnostics is available in Section 3 – “*Chromatographic peak models*”.


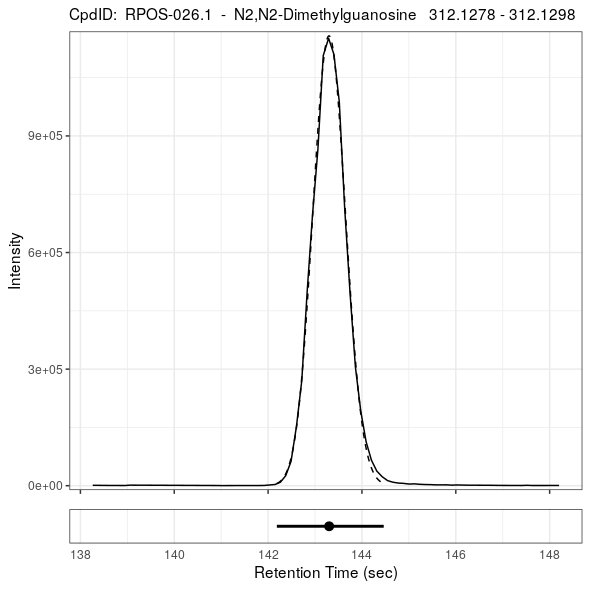


**Figure 3** – Extracted ion chromatogram from Figure 2 with the corresponding best fitting skewed gaussian chromtographic peak-shape model (dashed black line). The 95% retention time boundaries and apex of the fitted peak are shown in the bottom rectangular panel (black line with a dot).

The curve fitted models are then used to estimate a set of summary parameters for each ROI per each sample, which are described in *Table 2*.

| Metric | Interpretation | Formula (PASS/FAIL curve-fitting) |
| --- | --- | --- |
| peakArea | Signal integral estimated using the line-shape model | $\int_{rt=rtMin}^{rt=rtMax} f\left( rt;\theta\right)$/ (See *Table 4*) |
| peakAreaRaw | Signal integral calculated using the raw data points falling within the rtMax and rtMin boundaries. | $\int_{rt=rtMin}^{rt=rtMax} {intensity}_{rt}$/ (See *Table 4*) |
| maxIntMeasured | Maximum observed signal intensity | $max\left( intensity \right)$/ (See *Table 4*) |
| maxIntPredicted | Intensity at peak apex as estimated by the line-shape model | $max\left( f\left( rt;\theta\right) \right)$/ NA |
| mz | Accurate m/z estimation for the peak | $\frac{\sum_{i=1}^{n=N_{scans}} {intensity}_{i}\times{mz}_{i}}{{intensity}_{i}}$ / (See *Table 4*) |
| rt | Position of the peak apex (in RT) on the fitted curve (maxIntPredicted) | $rt\left[ argmax\left( f\left( rt;\theta\right) \right) \right]$ / (See *Table 4*) |
| mzMin | Smallest m/z value where intensity is larger than 0 within the elution time of the fitted line-shape (rtMin - rtMax) | $min\left( mz\left[ rt>rtMin\wedge rt<rtMax \right] \right)$/ (See *Table 4*) |
| mzMax | Largest m/z value where intensity is larger than 0 within the elution time of the fitted line-shape (rtMin - rtMax) | $max\left( mz\left[ rt>rtMin\wedge rt<rtMax \right] \right)$/ (See *Table 4*) |
| rtMin | Leftmost (peak front) boundary of the chromatographic peak. Estimated as the first retention time value where the intensity of the fitted peak reaches 0.5% of maxIntPredicted. | Found by interpolating the retention time value corresponding to an intensity of 0.05% maxIntPredicted on the peak front/ (See *Table 4*) |
| rtMax | Rightmost (peak tail) boundary of the chromatographic peak. Estimated as the second retention time value where the intensity of the fitted peak drops to 0.5% of maxIntPredicted. | Found by interpolating the retention time value corresponding to an intensity of 0.05% maxIntPredicted on the peak tail / (See *Table 4*) |
| isFilled | True if the values were estimated using Fallback Integration, false otherwise. | FALSE / TRUE |
| isFound | True if there was a signal in the EIC, false otherwise. | TRUE / TRUE |
| peakAsymmetry | Peak asymmetry measure. The front (rt_f_) and back (rt_b_) slopes are estimated as the points where the peak height is 10% of maxIntPredicted. PeakAsymmetry is the ratio of the distances between slopes at 10% peak height and the apex position (peak centre). | $\frac{{rt}_{t}-rt}{rt-{rt}_{f}}$/ NA |
| tailingFactor | Peak tailing measure. The front (rt_f_) and tail (rt_t_) slopes are estimated as the points where the peak height is 5% of maxIntPredicted. The tailingFactor is the ratio of the distance between the slopes to twice the distance from peak apex to tail slope. | $\frac{{rt}_{t}-{rt}_{f}}{2\left( rt-{rt}_{f} \right)}$/ NA |
| ppmError | m/z measurement error in part per million (ppm) | $\frac{mz-{ROI}_{mz}}{{ROI}_{mz}}\times1e6$ |
| rt_dev_sec | Difference between the original “rt” value provided in the ROI and the rt position of the peak apex | $rt-{ROI}_{Rt}$ |

**Table 2** – Summary statistics estimated for each targeted feature in individual samples and their respective calculation formulas. These statistics rely on a fitted peak-shape model. If the fit is not accepted (FAIL condition in the 3^rd^ column), then fallback integration region (FIR) is applied instead, and equivalent metrics are estimated as shown in Table 4. In the formula column, $f$() is the chromatographic peak-shape model function and θ is the vector of best fitting parameters found during the curve-fitting process.

**Generation and Output of Summary Statistics (*annotationParamsDiagnostic; outputAnnotationDiagnostic; outputAnnotationResult*):**

After running a peakPantheR annotation, the *annotationParamsDiagnostic* command can be used to process the results from the annotation and calculate summary statistics over all samples for all targeted features. It returns a modified *peakPantheRAnnotation* object:

updated_annotation <- annotationParamsDiagnostic(data_annotation, verbose=TRUE)

The *annotationParamsDiagnostic* method described above generates new uROI and FIR information from the results of a previously annotated *peakPantheRAnnotation* object.

These regions are estimated by pooling together the successful results from the initial annotation call (ROIs from samples where signal was found and an acceptable peak-shape model was fitted). The objective is to automatically calculate a consensus ROI which is broad enough in retention time and m/z to capture the signals as they are observed across multiple samples, without requiring the user to manually adjust ROI boundaries, except in more complex cases (misannotation, signal overlap, *etc)*. The new mzMin and mzMax values for uROI and FIR are set equal to the smallest and largest mzMin and mzMax estimated from all the successful annotations, respectively. The new retention time window values rtMin and rtMax are estimated in a similar manner, but each value is extended by +/- 5% of the total width (rtMax-rtMin). These rules are used to generate a new table with ROI information, similar to the one shown in ***Table 3***:

| CSV Column Name | Field meaning | Example entry | Origin |
| --- | --- | --- | --- |
| cpdID | Unique identifier for the MS feature | RPOS-026.1 | Defined by user in initial CSV input |
| cpdName | Text field with the name of the compound (does not need to be unique) | N2, N2- Dimethylguanosine | Defined by user in initial CSV input |
| ROI_mz | Expected m/z value (Theoretical m/z) | 312.1302 | Defined by user in initial CSV input |
| ROI_mzMin | Lower expected bound for m/z | 312.1256 | Defined by user in initial CSV input |
| ROI_mzMax | Upper expected bound for m/z | 312.1349 | Defined by user in initial CSV input |
| ROI_rt | Expected retention time (rt) | 143.22 | Defined by user in initial CSV input |
| ROI_rtMin | Lower expected bound for rt | 138.22 | Defined by user in initial CSV input |
| ROI_rtMax | Upper expected bound for rt | 148.22 | Defined by user in initial CSV input |
| uROI_mzMin | Lower estimated bound for m/z | 312.1261 | Estimated with *annotationParamsDiagnostic* |
| uROI_mzMax | Upper estimated bound for m/z | 312.1347 | Estimated with *annotationParamsDiagnostic* |
| uROI_rtMin | Lower estimated bound for rt | 141.5 | Estimated with *annotationParamsDiagnostic* |
| uROI_rtMax | Upper estimated bound for rt | 145.5 | Estimated with *annotationParamsDiagnostic* |
| FIR_mzMin | Same as uROI_mzMin, but used in FIR | 312.1261 | Estimated with *annotationParamsDiagnostic* |
| FIR_mzMax | Same as uROI_mzMax, but used in FIR | 312.1347 | Estimated with *annotationParamsDiagnostic* |
| FIR_rtMin | Same as uROI_rtMin, but used in FIR | 141.5 | Estimated with *annotationParamsDiagnostic* |
| FIR_rtMax | Same as uROI_rtMax, but used in FIR | 145.5 | Estimated with *annotationParamsDiagnostic* |

**Table 3** – Example ROI, uROI and FIR entries for the *[M+H]^+^* ion from N2, N2- Dimethylguanosine, for a reversed-phase positive mode LC-MS assay. The ROI entries are provided by the user in the initial input to *peakPantheR*. uROI and FIR suggestions are automatically estimated using the results of a previously annotated *peakPantheRAnnotation* object and can be further refined by the user.


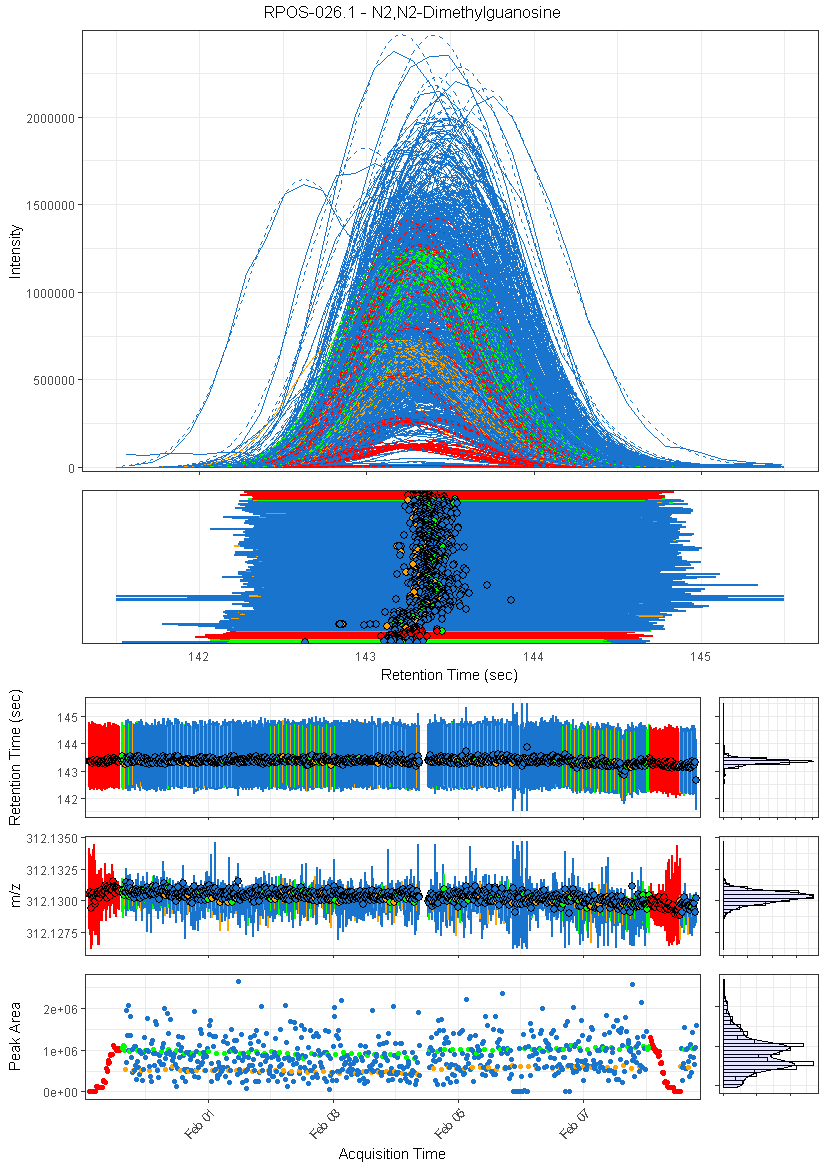


**Figure 4** – Example ([M+H]^+^ ion from N2, N2- Dimethylguanosine reversed-phase positive mode LC-MS assay) output plot generated for each ROI by the outputAnnotationDiagnostic function. Individual samples are colored based by information provided by the user. In this example, study samples are represented in blue, repeated injections of pooled study reference in green, repeated injections of a long-term reference sample in orange, and dilution series of the study reference in red (see Section 7 for details).

The results from a finalized annotation can be plotted via the *outputAnnotationDiagnostic* method on the updated *peakPantheRAnnotation* object containing the calculated summary metrics:

[outputAnnotationDiagnostic](https://phenomecentre.github.io/peakPantheR.github.io/reference/outputAnnotationDiagnostic-peakPantheRAnnotation-method.html)(updated_annotation, saveFolder=”./annotation_plots/”, savePlots=TRUE, verbose=TRUE, ncores=2, sampleColour=colorVector)

Where sampleColour is a vector of R colour specifiers one for each sample in the *updated_annotation*. Upon executing the *outputAnnotationDiagnostic* one figure per target feature will be generated and saved in the *saveFolder* directory. An example diagnostic plot is shown in ***Figure 4***. Each figure is comprised of five subplots displaying different aspects of the diagnostics across all samples, with data in each coloured according to sampleColour specifier. The first subplot contains the raw extracted ion chromatograms (solid line) with the corresponding line-shape fits (dashed line) for each sample. Immediately below there is a skyline projection of the detected peaks, where each is represented as a line in a fixed y-axis (run order) value from rtMin to rtMax in the x-axis (retention time), with a solid dot marking the peak apex (estimated as shown in ***Table 2*** and ***Table 4***). The next three subplots display the retention time, m/z and integrated peak area across the entire timescale of sample acquisition, sorted by run order, with each point on the X-axis representing a sample. The lines and dots plotted in the retention time panel represent the same as in the skyline figure above. For the m/z subplot, the lines represent the mzMin-mzMax boundary and m/z value measured (see ***Table 2*** and ***Table 4*** for the calculation details). The Peak Area panel contains a dot representing the integrated area measurement per sample, the peakArea parameter. Samples can be coloured according to any criteria by passing a vector of colours with a number of elements equal to the number of samples. These plots are designed to assist in identifying trends in intensity, m/z and retention time shift with run order, which can subsequently guide the re-parametrisation of the ROI windows for a subsequent round of annotation. Besides the figures, a CSV file ‘*annotation_summary.csv’* is also generated. This file contains the regions of interest used as well as uROI and FIR suggestions estimated from the previous annotation process, and resembles the output shown in ***Table 3***. This file should be modified and re-loaded to sequentially adjust the integration boundaries for each target feature.

The *outputAnnotationResult* will save a series of .CSV files containing the values for all the parameters shown in ***Table 2*** and ***Table 4***, including matrices detailing the presence or absence of a signal in a sample (isFound and isFitted).

outputAnnotationResult(updated_annotation,saveFolder=’./annotation.’,annotationName=’example’, verbose=TRUE)

**Updating Annotations from the uROI/FIR (*resetAnnotation*):**

In the standard *peakPantheR* workflow, the uROI and FIR will be automatically estimated after running *peakPantheR* with a set of ROI as shown in ***Table 3*** and saved as a csv using *outputAnnotationDiagnostic*. The annotation can subsequently be updated, applying the uROI and FIR regions by loading this csv file and running *resetAnnotation*:

newAnnotation <- peakPantheR_loadAnnotationParamsCSV(csvFile)

newAnnotation <- resetAnnotation(newAnnotation, *spectraPaths*=files_all,

*spectraMetadata=spMetadata*, u*ROI*=TRUE, *useFIR*=TRUE)

Prior to this point the uROI and FIR regions can be checked using the visual outputs and amended manually in the case of any mis-annotations or signal overlaps before running *resetAnnotation*.

It is also worth noting that it is possible to start directly from a set of uROI and FIR values or upload the CSV outputs from a previous annotation:

newAnnotation <- peakPantheR_loadAnnotationParamsCSV(csvFile)

newAnnotation <- resetAnnotation(newAnnotation, *spectraPaths*=files_all,

*spectraMetadata=spMetadata*, u*ROI*=TRUE, *useFIR*=TRUE)

newAnnotation_results <- peakPantheR_parallelAnnotation(newAnnotation, ncores=4,

resetWorkers=30,verbose=TRUE)

The useFIR argument defines whether the fallback integration functionality should be used in case of failure to fit a valid line shape, while the uROI argument specifies whether to search for the features in the uROI or revert to the original ROI values. The *peakPantheR_loadAnnotationParamsCSV* method initialises an object containing only the ROI/uROI/FIR values used and the *resetAnnotation* method is used to re-set values in the *peakPantheRAnnotation* object. In the above example, it is being used to configure the list of files to read *spectraPaths,* their metadata (*spectraMetadata*), and the useFIR and uROI settings.

The purpose of fallback integration is to account for cases where the curve-fitting procedure failed or resulted in a rejected line-shape, with a high residual deviation from the raw EIC data points. This can be due to deterioration of peak-shape, insufficient signal digitisation, signal saturation or low signal to noise ratio. It is only performed if the argument *useFIR* is set to TRUE, otherwise all outputs are returned as NA. The main purpose of this step is to obtain an estimate of the noise baseline when no signal is present or for the signal integral for peaks with very irregular line-shape that cannot be well described by classic gaussian-like chromatographic peak models, which are common in HILIC chromatography^16^. For consistency, it is recommended to define the FIR boundaries equal to those of the ROI/uROI. The FIR can be set to automatically match the uROI by using *resetFIR* method.

newAnnotation <- resetFIR(newAnnotation)

The fallback integration procedure calculates similar metrics as the curve-fitting process, but without using the parametric line-shape model. Since no chromatographic peak shape was obtained in the curve-fitting procedure, *peakAsymmetry*, *peakWidth* and *peakTailingFactor* cannot be calculated*,* and these are set to “NA”. The formulas used to estimate all other metrics with fallback integration are shown in ***Table 4***.

| Metric | Interpretation | Formula |
| --- | --- | --- |
| peakArea | Estimated total signal integral. Calculated using the trapezoid rule, by multiplying the average intra scan delay (scanRtDiff) by sum of the maximum intensity value observed at each scan (rtMaxIntensity) | $peakArea=trapezoid(\sum rtMaxIntensity\times scanRtDiff$  where $rtMaxIntensity=\sum_{i=i}^{n=N_{scans}} max\left( {intensity}_{i} \right)$ |
| peakAreaRaw | peakAreaRaw is the same as peakArea for fallback integration | Same as peakArea |
| maxIntMeasured | Maximum observed signal intensity | $max\left( intensity \right)$ |
| maxIntPredicted | Not applicable for FIR | NA |
| mz | Arithmetic mean of m/z values weighted by signal | $\frac{\sum_{i=1}^{n=N_{scans}} {intensity}_{i}\times{mz}_{i}}{{intensity}_{i}}$ |
| rt | Retention time value at signal maximum | $rt\left[ argmax\left( intensity \right) \right]$ |
| mzMin | Smallest m/z value where intensity is larger than 0 within the user defined region of interest | As defined in FIR settings |
| mzMax | Largest m/z value where intensity is larger than 0 within the user defined region of interest | As defined in FIR settings |
| rtMin | Smallest retention time value where intensity is larger than 0 within the user defined region of interest | As defined in FIR settings |
| rtMax | Largest retention time value where intensity is larger than 0 within the user defined region of interest | As defined in FIR settings |
| isFilled | Was fallback integration used? | TRUE or FALSE |
| isFound | Was there any signal in the ROI? | TRUE or FALSE |
| peakAsymmetry | Not applicable for FIR | NA |
| tailingFactor | Not applicable for FIR | NA |
| ppmError | Mass measurement error in part per million (ppm) | $\frac{mz-{ROI}_{mz}}{{ROI}_{mz}}\times1e6$ |
| rt_dev_sec | Difference between the original “rt” value provided in the ROI and the rt position of the peak apex | $rt-{ROI}_{Rt}$ |

**Table 4** – Fallback integration estimates for the summary statistics reported in Table 2.

**
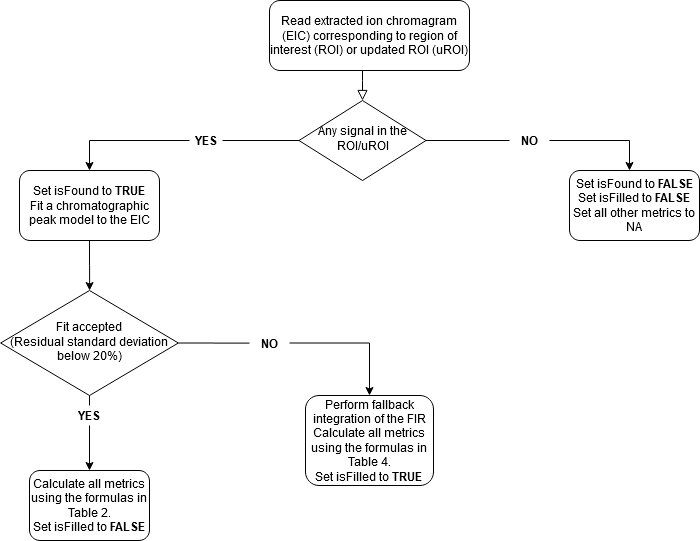
**

**Figure 5** - Possible outcomes of the peak detection, fitting and integration, and the corresponding state for the attributes in the peakPantherAnnotation object and in the .csv output files.

This process can be repeated (iterations of *resetAnnotation*, *outputAnnotationDiagnostic*, check and amend uROIs) until the user is satisfied with the integration results. Once no further modifications to the regions of interest are needed, the parameters and quality metrics can be exported as text files for further analysis with other software tools using the *outputAnnotationResult* method. For example, the outputs of peakPantheR are recognised by the nPYc-Toolbox^8^⁠, a python toolbox for quality control of metabolic profiling datasets. A basic tutorial on how to read and run peakPantheR integration results on the nPYc-Toolbox can be found here <https://github.com/phenomecentre/nPYc-toolbox-tutorials>. More examples of this functionality are also available in the github repository of the dementia study <https://github.com/phenomecentre/metabotyping-dementia-urine>.

So far, the functionality has been exemplified with the more complex case of integrating entire sample sets. PeakPantheR can be applied directly on a single file with the *peakPantheR_singleFileSearch* method. All the functionality showcased above is available, with the exception that it is not possible to automatically calculate summary statistics for the whole dataset and estimate uROI or FIR from previous fit results.

annotation <- peakPantheR_singleFileSearch(singleSpectraDataPath = input_spectraPath,

targetFeatTable = input_targetFeatTable,

peakStatistic = TRUE,

verbose = TRUE)

For a worked example of single file annotation, see the “Real-Time Annotation” vignette.

# Chromatographic peak models

Upon reading the raw data for a given ROI in a sample and assembling the extracted ion chromatogram (EIC), *peakPantheR* will attempt to fit a parametric line-shape representing the chromatographic peak. The default line-shape model is a skewed Gaussian peak-shape (Equation 1)^17^.

$$f\left( x;A,\mu,\sigma,\gamma\right)=\frac{A}{\sigma\sqrt{2\pi}}e^{\left[ \frac{-\left( x-\mu\right)^{2}}{{2\sigma}^{2}} \right]}\left[ 1+erf\left( \frac{\gamma\left( x-\mu\right)}{\sigma\sqrt{2}} \right) \right]$$

Equation 1 – Skewed Gaussian line-shape

where erf is the Gauss error function:$erf\left( x \right)=\frac{2}{\sqrt{\pi}}\int_{0}^{x} e^{-t^{2}}dt$, and is calculated by taking advantage of the identity $erf\left( x \right)=2P\left( x\sqrt{\pi} \right)-1$⁠^18^, where P(x) is the cumulative distribution function for the normal distribution.

This distribution allows for peak asymmetry and tailing, which are characteristic in chromatographic peaks. It is parameterized as shown in Equation 1. This model has a parameter for the peak position (µ), amplitude (A), peak-width (δ) and peak skew (γ). Only one line-shape is fitted per ROI, as it is expected that a single well-defined chromatographic peak exists in the integration region. The current version of peakPantheR (v1.4.0) does not provide multiple signal deconvolution features.

Another available line-shape is the exponentially modified Gaussian.

$$f\left( x;A,\mu,\sigma,\gamma\right)=\frac{A\gamma}{2}e^{\left[ \gamma\left( \mu-x+\frac{\gamma\sigma^{2}}{2} \right) \right]}erfc\left( \frac{\mu+\gamma\sigma^{2}-x}{\sqrt{2}\sigma} \right)$$

Equation 2 – Exponentially modified Gaussian (EMG) line-shape

where erfc is the complementary Gauss error function:$erfc\left( x \right)=1-erf\left( x \right)$

The starting solution for the curve-fitting minimization problem is estimated from the extracted ion chromatogram and constrained using the generated parameters as shown in ***Table 5***.

| **Parameter** | **Guess method estimate** | **Lower bound** | **Upper bound** |
| --- | --- | --- | --- |
| Amplitude (A) | 1e7 | 0 | 1e9 |
| Peak position (µ) | $rt\left[ argmax\left( intensity \right) \right]$ | µ guess - 3 (s) | µ guess + 3 (s) |
| Peak-width (δ) | 1 (s) | 0 | 5 |
| Skew (γ). | 1 | -0.1 | 5 |

**Table 5** – Initial estimates for the peak-shape model parameters.

The curve-fitting procedure is performed using the *nls.lm* function from the minpack.lm^11^⁠ package. This package provides a high-level R interface for the Levenberg-Marquardt routines in the MINPACK numerical library^19^⁠. Minpack.lm has a series of built-in numerical convergence checks to flag lack of solver convergence and other optimization errors. If the fit *nls.lm* function finishes with such an error message or warning, the extracted ion chromatogram is evaluated using the fallback integration rules described in ***Table 4***. If the curve-fitting procedure converges successfully to a minimum, the obtained best-fitting line-shape is subjected to a further series of quality control diagnostics. The total line-shape residual and the residual deviation at the peak apex must not exceed 20% of the value in the raw data EIC. Failing these criteria leads to rejection of the fit and evaluation of the EIC using the fall back integration (see ***Figure 5***). The fitted line-shape is then used to calculate the summary peak statistics shown in ***Table 2***.

Selection of the line-shape model to fit is performed through the *curveModel* argument of the *peakPantheR_singleFileSearch* and *peakPantheR_parallelAnnotation* methods. Allowed values are ‘*skewedGaussian*’ (default), for the skewed Gaussian model, and *‘emgGaussian’* for the exponentially modified Gaussian.

annotation <- peakPantheR_singleFileSearch(singleSpectraDataPath = input_spectraPath,

targetFeatTable = input_targetFeatTable,

peakStatistic = TRUE, curveModel=’emgGaussian’,

verbose = TRUE)

newAnnotation_results <- peakPantheR_parallelAnnotation(newAnnotation,

ncores = 10, resetWorkers = 30,

curveModel = ‘emgGaussian’, verbose = TRUE)

# Retention time adjustment

The purpose of the retention time adjustment models in *peakPantheR* is to adjust the retention time centre and boundaries of the ROIs to reflect between-experiment retention time deviations and other chromatographic batch effects**.** Unlike the *m/*z information, for which theoretical values can be calculated from chemical formulas, retention time values are always empirical measurements. When building a retention time database from either standards or previous observations of a compound in a sample matrix, the recorded retention time values depend on experimental conditions at the time of data acquisition. When the same methodology is applied to new sample sets, the entire retention time reference grid from the database will need some degree of correction, to reposition the expected retention times for each feature. This only needs to be done once at the beginning of the workflow, as progressive sample to sample retention time deviations are accounted for by setting appropriate retention time windows for each ROI.

Retention time correction can be applied to a list of ROIs using peakPantheR’s *retentionTimeCorrection* method. Briefly, the initial set of uncorrected ROIs are read and annotated to obtain a *peakPantheRAnnotation* object with their observed retention time values in the dataset. The set of results derived from this initial annotation will be then used to calculate the retention time deviations from the expected ROI value:

${Rt}_{Drift}={Rt}_{ROI}-{Rt}_{Observed}$

The measured *RtDrift* is calculated by *peakPantheR* by default (*rt_dev_sec* metric mentioned in ***Table 2*** and ***Table 4***), and can be used to fit a retention time correction function, which models retention time drift as a function of the expected “true” retention time value. The expected retention time value is taken from the ROI_rt if the *peakPantheRAnnotation* attribute *useUROI* is set to FALSE, otherwise from uROI_rt. The expected ROI rt values can then be corrected by subtracting the estimated *RtDrift* from the default value.

$\hat{{Rt}_{Drift}}=f\left( {Rt}_{ROI} \right){Rt}_{Corrected}={Rt}_{ROI}-f\left( {Rt}_{obs} \right)$

The *retentionTimeCorrection* method supports the fitting of polynomial and (*method=’polynomial`*), linear (*method=’polynomial`* and *params=list(polynomialOrder=1)*) functions as well as constant offset substraction (*method=’constant’*), for cases where a single retention time reference is available.

rtCorrectedAnnotationPolynomial <- retentionTimeCorrection(method=’polynomial’, params=list(polynomialOrder=3), robust=F, rtWindowWidth=15, rtCorrectionReferences=NULL)

rtCorrectedAnnotationLinear <- retentionTimeCorrection(method=’polynomial’, params=list(polynomialOrder=1), robust=F, rtWindowWidth=15)

rtCorrectedAnnotationConstant <- retentionTimeCorrection(rtCorrectionReferences=c(‘Cpd-1’), method=’constant’, robust=F, rtWindowWidth=15)

The references used for retention time correction must be previously annotated features, and can be selected by passing a vector of *‘CpdID’* entries. By default *CorrectionReferences=NULL*, which triggers usage of all features as reference. The *rtWindowWidth* argument controls the width of the uROI and FIR retention time windows after correction (rtMax-rtMin). rtMin and rtMax are calculated as rt ± *rtWindowWidth*/2, where rt is the adjusted retention time value.

One limitation with this correction procedure is that it assumes that the annotated signals on the first ROI pass correspond to the correct reference. In the presence of more severe retention time drifts, some of the compounds will be misannotated, and will influence negatively the estimation of the correction function. To improve the robustness of the retention time correction procedure, we have implemented a RANSAC^20^⁠ retention time fitting option in peakPantheR. Briefly, the RANSAC algorithm is used to infer automatically if any of the data points (pair of expected retention time and measured drift in seconds) is likely to be an outlier, and automatically exclude it from the function fitting procedure. It works by repeatedly fitting the function of choice (linear or polynomial) multiple times to a random subset of the data and estimating the median residual deviation per data point across all random samples. Points which have a high median residual deviation are labelled as outliers and not used to estimate the final best-fitting retention time correction function parameters. We do not recommend using the RANSAC fitting option with a small number of compounds (< 10). The method’s performance improves with the number of non-coeluting targets, as it becomes easier to differentiate the main retention time deviation trend which needs to be corrected from extreme outlying deviations caused by misannotation.


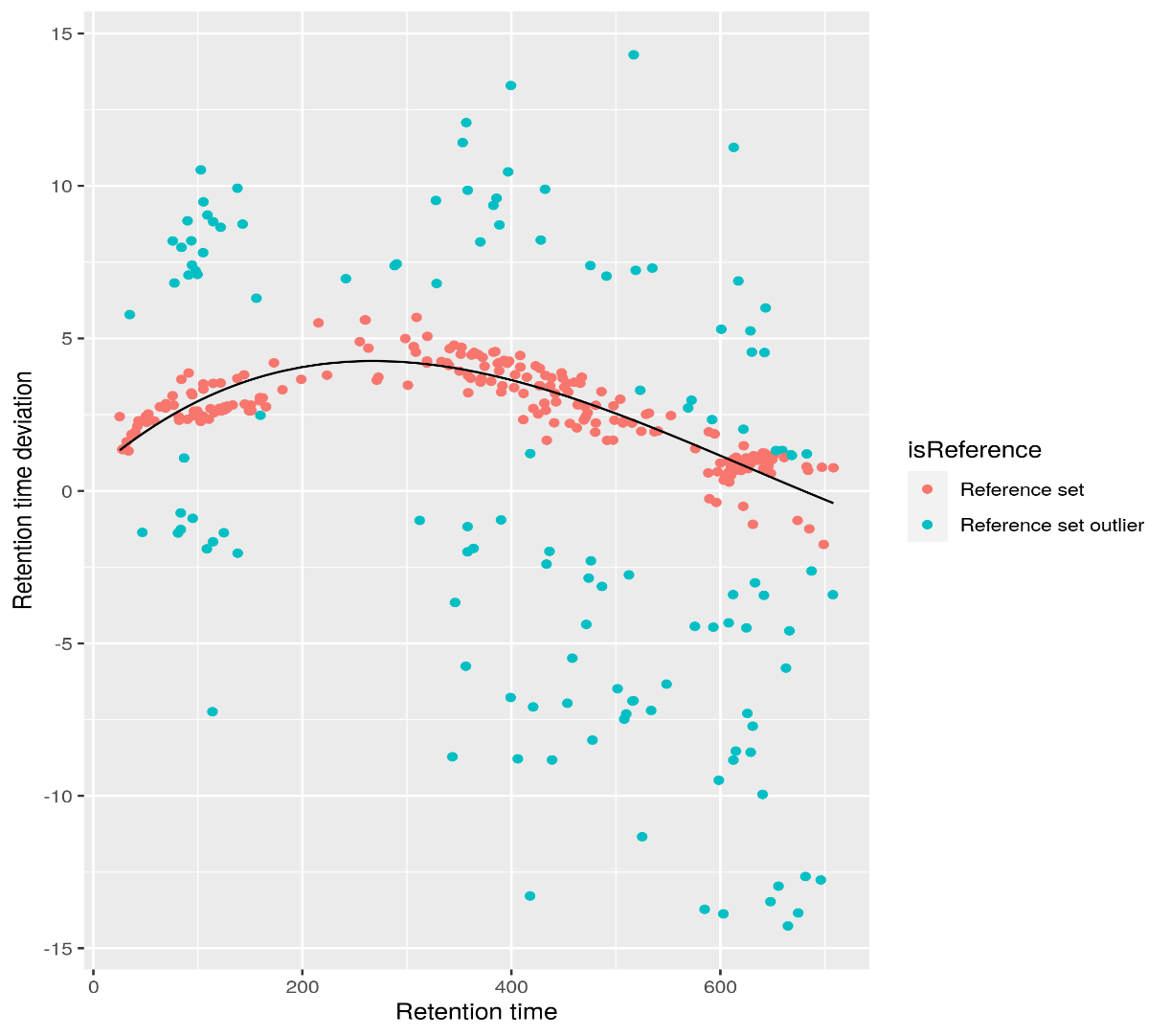


**Figure 6** – Example diagnostic plot generated by the retentionTimeCorrection method. The observed retention time deviation is shown (y-axis) as a function of the expected retention time (x-axis). A 3^rd^ degree polynomial was fitted with the RANSAC algorithm to obtain the retention time correction function (solid black line). Features colured in blue (‘Reference set outlier’) were determined with RANSAC to be potential outliers, and excluded from the dataset before finding the best fitting parameters for the retention time correction function. Conversely, all features coloured in red (‘Reference set’) were used to fit the final correction function.

To apply the RANSAC algorithm to “robustify” the calibration function fit, set robust=TRUE on the retentionTimeCorrection method call:

rtCorrectionOutput <- retentionTimeCorrection(method=’polynomial’, params=list(polynomialOrder=3), robust=TRUE, diagnostic=TRUE)

Running the retention algorithm will return a new *peakPantheRAnnotation* object and a diagnostic plot such as the one shown in ***Figure 6*** (if *diagnostic=FALSE,* no plot will be returned):

rtCorrectedAnnotation <- rtCorrectionOutput$annotation

diagnosticPlot <- rtCorrectionOutput$plot

The *peakPantheRAnnotation* object returned is a copy of the original object, but with modified uROI and FIR retention time information (uROI_rt, uROI_rtMin, uROI_rtMax, FIR_rtMin and FIR_rtMax). No *m/z* fields are modified. The *useFIR* and *useUROI* attributes in the output *peakPantheRAnnotation* object are also set to TRUE.

If the quality of the calibration function fit is acceptable (judging from the diagnostic plot), the new *peakPantheRAnnotation* object is ready for use and feature extraction on the retention time corrected windows can be performed straight away using the *peakPantheR_parallelAnnotation* function. We recommend manually reviewing the goodness-of-fit of the retention time correction function before proceeding to the targeted annotation with the corrected uROI. The RANSAC algorithm is non-deterministic and can generate a different output every time it is executed, even with the default parameters.

# Using peakPantheR to integrate features from a large LC-MS dataset: examples and recommendations:

In this section, we exemplify how peakPantheR can be used as a targeted peak-picker to integrate hundreds of compounds in a typical LC-MS dataset. A series of tutorial scripts are available via a GitHub repository: <https://github.com/phenomecentre/metabotyping-dementia-urine>. These cover integration of pre-annotated compounds in a large human (approximately 650 samples) urine biofluid dataset, profiled using three LC-MS assays (Reversed-phase positive mode, reversed-phase negative mode and HILIC-positive mode)^7^. Due to the large file size, the mzML files used in this examples need to be downloaded separately from MetaboLights: <https://www.ebi.ac.uk/metabolights/MTBLS719/>. The steps detailed in this section can be applied to integrate any of the LC-MS assays, and separate scripts and compound annotation files to do so are provided in the git repository.

The acquisition of these LC-MS datasets followed the experimental design and acquisition procedure described in Lewis et al^7^. The main sample types and their purpose are described in ***Table 6***. ***Figure 7*** showcases the data acquisition and batch structure. In this figure, single data points correspond to the intensity of the same feature (y-axis) in individual samples, with the run order shown in the x-axis. Each analytical batch is bracketed by a serial dilution series (red) of the study reference sample (green). After the first dilution series, there are five injections of the study reference, followed by injection of the study samples (in blue). Study reference and long term reference (orange) samples are injected alternatedly every five study samples, until the end. The termination sequence is the reverse of the starting sequence, with 5 SR injections, and another serial dilution series of the SR injected in descending order.

| **Sample type** | **Sample type code** | **Color convention in examples** | **Description** |
| --- | --- | --- | --- |
| Study Reference | SR | Green | Study-specific pooled quality control sample. Created by pooling together a small volume of each study sample in the study. |
| Long Term Reference | LTR | Orange | Pooled urine biofluid sample, used across multiple projects. Generated separately by pooling multiple human urine samples, but does not contain material from the samples in this study. |
| Study Samples | SS | Blue | Individual study samples to be analysed. |
| Serial Dilution samples | SRD | Red | Serial dilutions of the SR sample. |

**Table 6** – Sample types present in the example LC-MS datasets, and their experimental purpose.


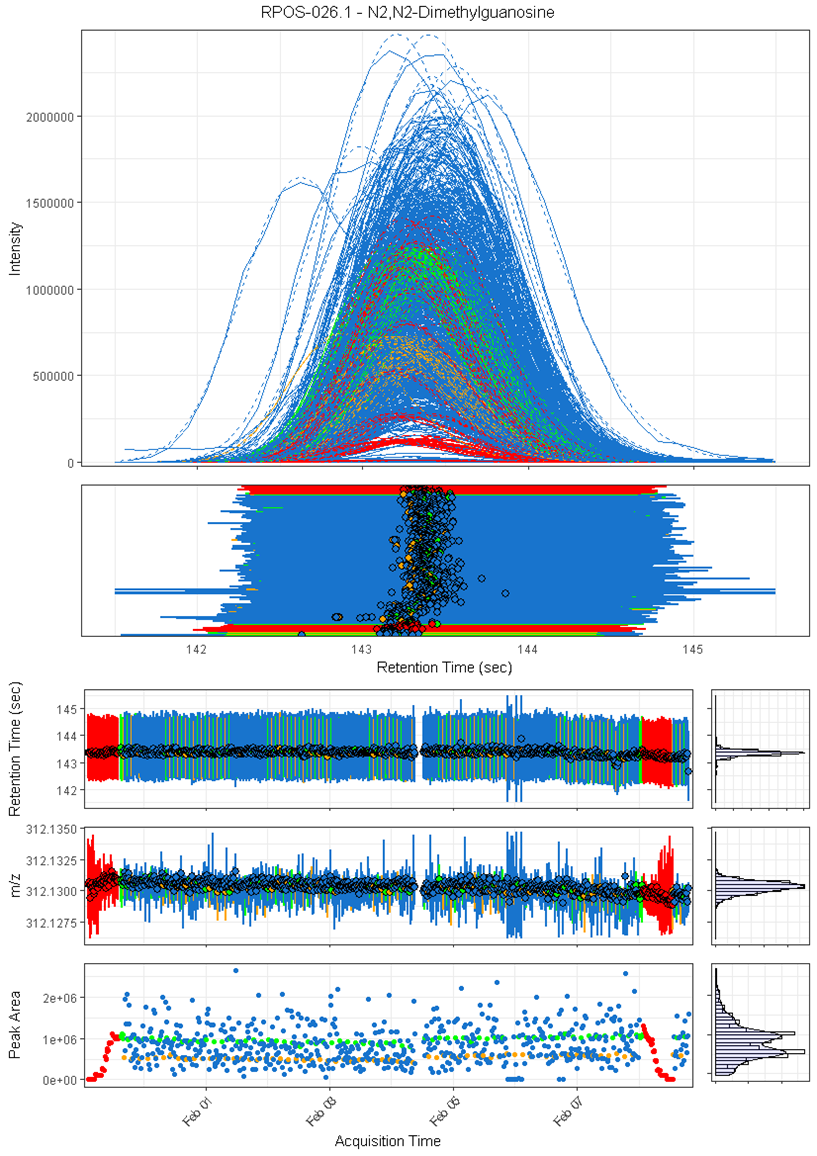


**Figure 7** – LC-MS analytical data acquisition sequence, exemplified using the intensity values (y-axis) of a randomly selected compound. Values are sorted by their run order (Acquisition Time). Study reference (green) and long term reference (orange) samples are injected every 5 study samples (in blue). A small number of samples were acquired after the final dilution series (re-runs). Those were re-injections of samples were data acquisition failed during the run and samples flagged previously by ^1^H NMR as containing contaminants or large amounts of protein.

The *parse_IPC_MS_project_names* function bundled with the example scripts will automatically parse the file names and assign each .mzML file to one of the sample types specified in ***Table 6***. This function facilitates subsetting of the data files when running peakPantheR workflows:

project_files <- parse_IPC_MS_project_names(rawData_folder, 'U')

files_all <- project_files[[1]]

metadata_all <- project_files[[2]]

The *metadata_all* dataframe contains the information of sample type, which is required to subset samples:

which_QC <- metadata_all$sampleType %in% c('LTR', 'SR')

**Setting up initial ROI parameters:**

Successful deployment of peakPantheR workflows requires the definition of comprehensive ROI/uROI/FIR regions which are specific for the ions of interest, but still broad enough to track the positional variation in both *m/z* and retention time across the whole experiment. Knowledge about the expected performance of the specific chromatographic method and mass spectrometer instrument should be used to set the starting ROI values.

The expected *m/*z value in the ROI should be set as the theoretical mass-to-charge ratio value for a given ion. The experimentally observed *m/z* value at the peak apex depends on the MS instrument’s mass accuracy, but to capture the entire peak, the *m/z* window (*mzMax* – *mzMin*) should match the instrument’s mass resolution as well. If external and internal calibration *m/z* calibration procedures are followed thoroughly, the *m/z* position at the peak apex can be assumed to vary randomly both within and between studies. The initial ROI window can then safely be set by centring it on the theoretical *m/z* value for a given ion and choosing *mzMax – mzMin* according to the instrument’s resolving power, expressed in Δ(*m/z*).

For the chromatographic retention time domain the situation is slightly more complex, as significant systematic deviations are expected both between and within experiments. Within a run, the retention time drift manifests as very small (less than one second) deviations in the peak apex position between samples consecutive in run order. This drift can be accommodated by allowing the chromatographic width of the integration windows to cover this variation. We suggest setting the default *rtMin* and *rtMax* values based on pre-existing information about the chromatographic gradient performance and the retention time position of the compound in some of the first and last quality control samples ran, respectively.

Between experiment deviations can pose major hurdles for signal integration and lead to compound misannotation and integration of the wrong signals. peakPantheR provides specific retention time adjustment functionality to deal with this problem. Details on these retention time adjustment procedures can be found in Section 4 – “*Retention time adjustment*”. In peakPantheR, these are applied after an initial run with the default ROI values, and the observed deviations used to fit correction functions and update the uROI.

The starting ROI information for the compounds in this example can be found in the git repository, under ‘./peakPantheR/LC-MS Annotations/ROI Files/RPOS_ROI.csv’. More information about the chemical compounds mentioned in the ROI files can be found in the ‘LC-MSAnnotations_peakPantheR.xlsx’ file. The ROI files already contain adequate mzMin/mzMax and rtMin/rtMax default values. The *m/z* window was set to theoretical *m/z* ± 30 ppm, to cover mass accuracy drifts and the instrumental mass resolution expected from the Time-of-Flight detector instrument used (Waters Xevo G2-S oaTOF). The retention time parameters were set based on the expected retention time for the chromatographic methods (*ROI_rt*) ± 5 seconds.

**Initial run of peakPantheR:**

To facilitate revision, we suggest running peakPantheR initially only on the repeated injections of the LTR and SR quality control samples:

data_annotation_wideWindows <- peakPantheRAnnotation(spectraPaths=files_all[which_QC], targetFeatTable=ROI, spectraMetadata=metadata_all[which_QC, ])

Since the SR and LTR quality control samples are acquired repeatedly throughout the study, their integration is sufficient to gauge if the initial ROI requires adjustement. This initial processing is saved as “wideWindows” in the examples. After this first run, the *annotationParamsDiagnostic* diagnostic method will suggest automated adjustments to the ROI based on successfully fitted peaks on the first run. We suggest re-running the *peakPantheR_parallelAnnotation* process with the “annotationParameters_summary.csv” obtained from updated “wideWindows” run:

update_csv_path <- file.path(work_dir, './wideWindows_annotation_SR_LTR/annotationParameters_summary.csv')

narrowWindows_annotation <- peakPantheR_loadAnnotationParamsCSV(update_csv_path)

narrowWindows_annotation <- resetAnnotation(narrowWindows_annotation, spectraPaths=files_all[which_QC], spectraMetadata=metadata_all[which_QC,], useUROI=TRUE, useFIR=TRUE)

narrowWindows_annotation_results <- peakPantheR_parallelAnnotation(narrowWindows_annotation, ncores=18, resetWorkers=30, verbose=TRUE)

We designate this second processing run as “*narrowWindows”* in the examples.


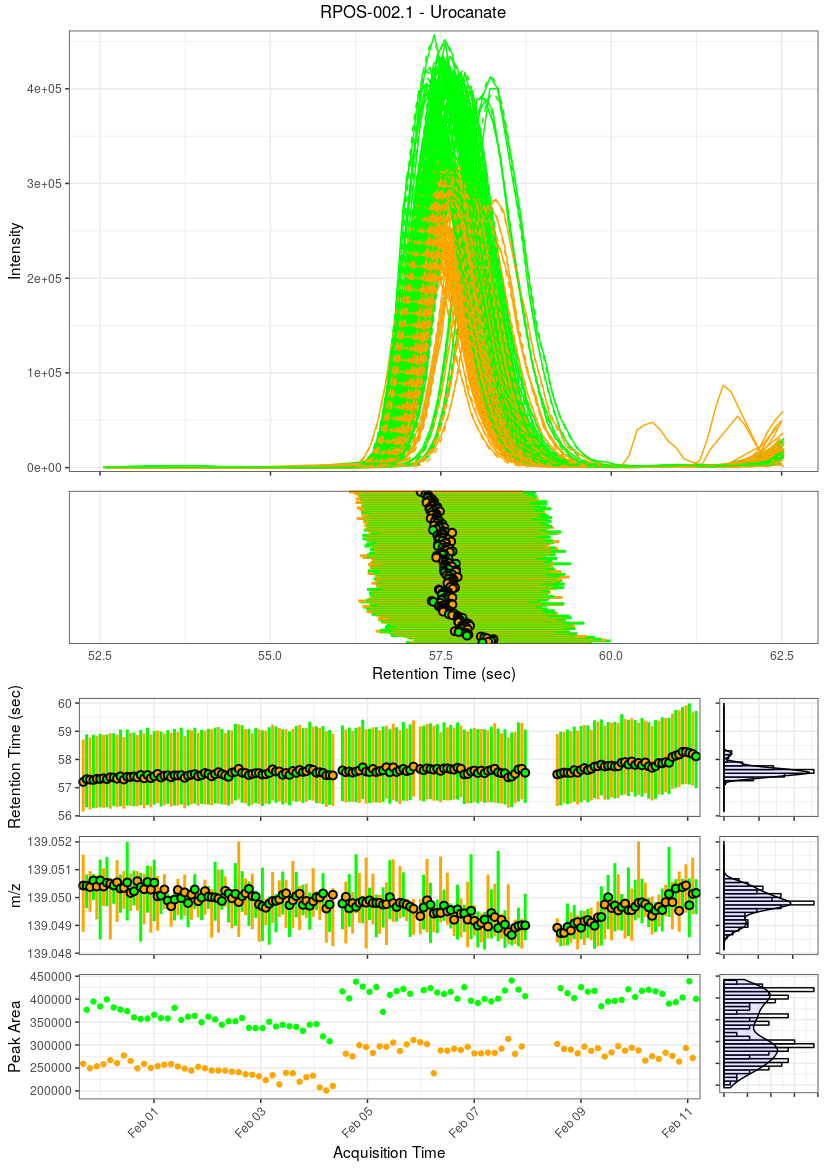


**Figure 8** – Diagnostic plot for Urocanate [M+H]^+^, for the initial “wideWindows” annotation results. Only Study reference (green) and long term reference (orange) injections were integrated. Since these samples are injected repeatedly across the study, the patterns of m/z and retention time variation observed are representative of the entire dataset.


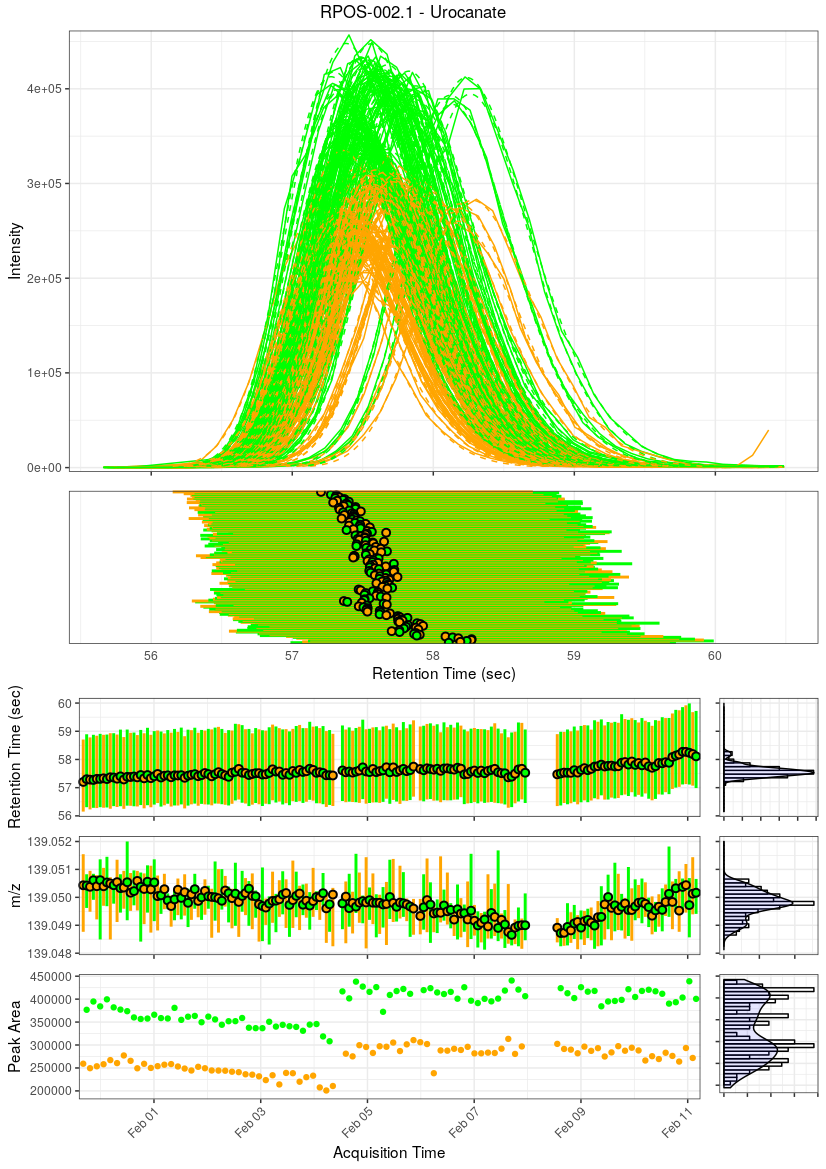


**Figure 9** - Diagnostic plot for the same ion shown in Figure 8, Urocanate [M+H]^+^, obtained from the “narrowWindows”annotation. The adjustments to the ROI were suggested automatically with the annotationParamsDiagnostic method.

While in this example this was not deemed necessary, for cases where retention time adjustment is needed, we suggest applying it using the information from the first “wideWindows” annotation and repeating the integration twice, first with the retention time corrected ROIs and then with the ROI refinements suggested by the *annotationParamsDiagnostic* from the previous run.

**Re-running and revision on entire dataset:**

Up until now, all adjustments to ROI/uROI were performed with minimal manual revision. At this stage it is recommended to visually inspect the figures from the “narrowWindows” annotation outputs. The diagnostic plots have been devised to facilitate revision of multiple chromatograms for a single feature at once, possibly the entire dataset. In practice, most of the iterative refinements of ROI/uROI/FIR parameters are done on the retention time boundaries (*rtMin/rtMax*). Manual adjustments to *mzMin/mzMax* are usually not required or restricted to pathological cases of analytical batches with *m/z* calibration issues, and tend to be applied to all features indiscrimately (e.g. increase *mzMax – mzMin* difference by a few ppm).


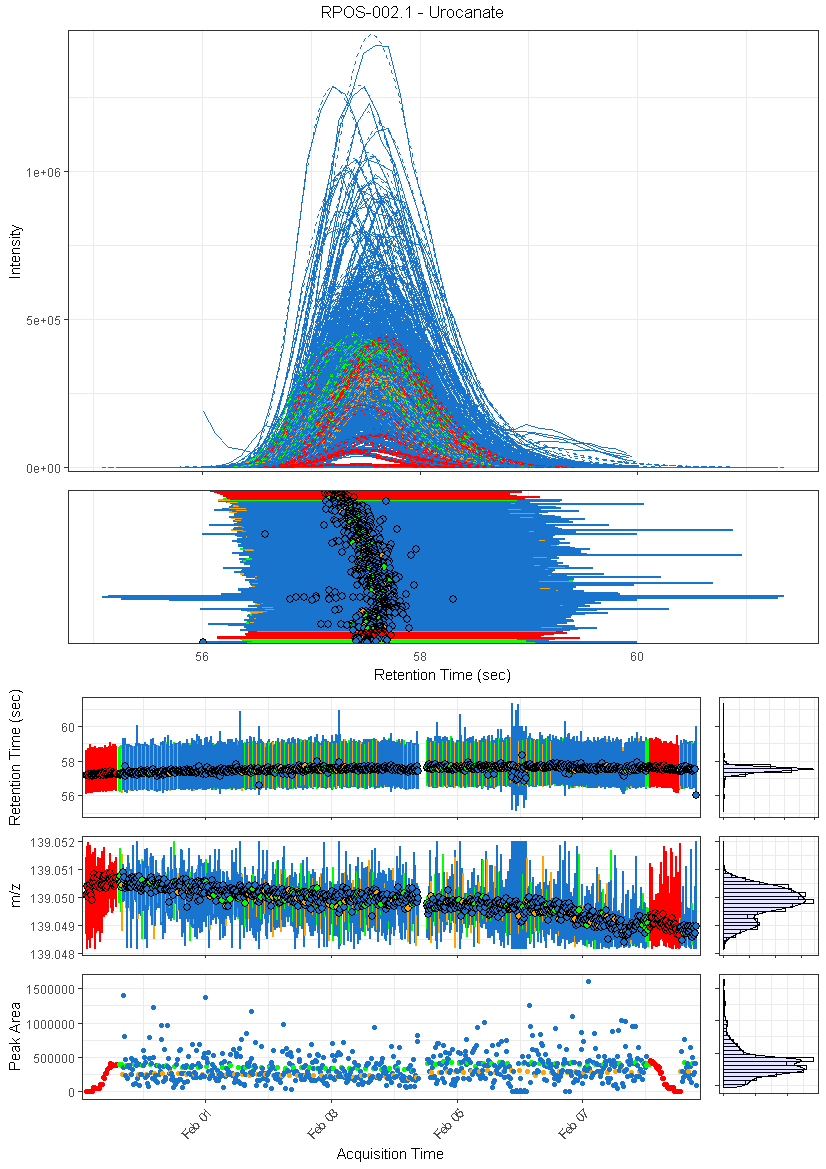


**Figure 10** - Diagnostic plot for the integration of Urocanate [M+H]^+^ in the entire dataset.

Once the integration boundaries look acceptable for the study reference and long term reference samples, the same parameters should be used to integrate all samples. The results can be iteratively reviewed and improved until the quality of the integration is acceptable. The example scripts also contain a step to save the results objects to .RData files. Revision can be performed with the help of the Shiny Graphical user interface. More details on how to use it are provided in Section 6 – Graphical user interface.

In addition, the results folder generated by o*utputAnnotationResult* method contains a series of .csv files with quality control metrics for all compounds across individual samples. Their overall distribution in the dataset or their trends with run order can be used in quality control workflows, and to flag specific compounds for manual revision.

# Graphical user interface

A Shiny graphical user interface is available to assist users in deploying peakPantheR for parallel annotation workflows. To start the graphical user interface, load the peakPantheR library and type the following command:

peakPantheR_start_GUI(browser=TRUE)

This will start the Shiny GUI on the default web browser. Setting ‘*browser=FALSE’* displays the app in an R plot tab on the RStudio IDE.

The graphical interface (***Figure 7***) is structured as a series of page tabs:

- **About:** This is the first landing page displayed when the GUI loads. It provides an overview of the peakPantheR package and its functionality.
- **Import Data:** Interface to initialize or import a *peakPantheRAnnotation* object.
- **Run:** Trigger the annotation of the compounds of interest across the specified files, either serially or in parallel.
- **Diagnostic: update and plots:** Interface to adjust the uROI/FIR parameters based on inspection of plots and results from a previous peakPantheR annotation run.
- **Integration results:** Displays the results and summary metrics from a *peakPantheRAnnotation* object in a tabular format.
- **Export results:** Export diagnostic plots, summaries and data matrices.


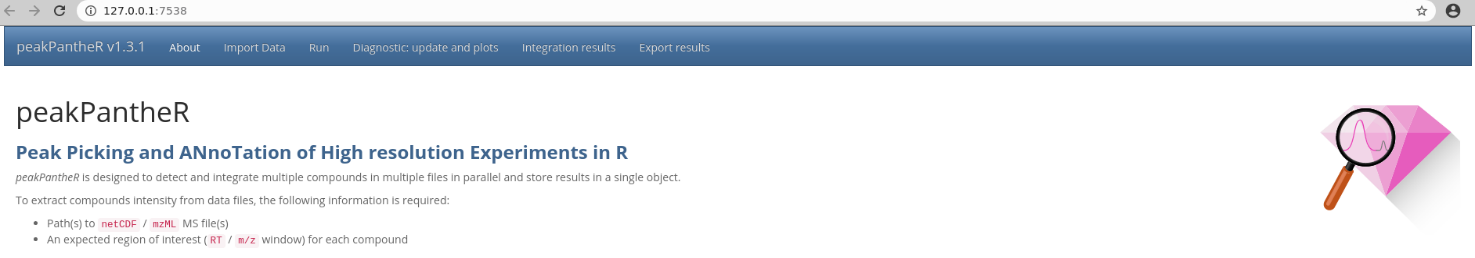


**Figure 11 –** Introductory **‘**About’ page from the peakPantheR ShinyGUI. Navigation to other tabs is performed via the top blue bar.

**Import data:**

The *‘Import Data’* tab (***Figure 8***) contains interactive forms to generate a new *peakPantheRAnnotation* object or import a pre-generated one. This *peakPantheRAnnotation* object is required before using any of the functionality in the other tabs. The import page is structured as 2 vertical rectangular panels, ‘*Create a new peakPantheRAnnotation*’ and *‘Load a peakPantheRAnnotation’*, respectively.


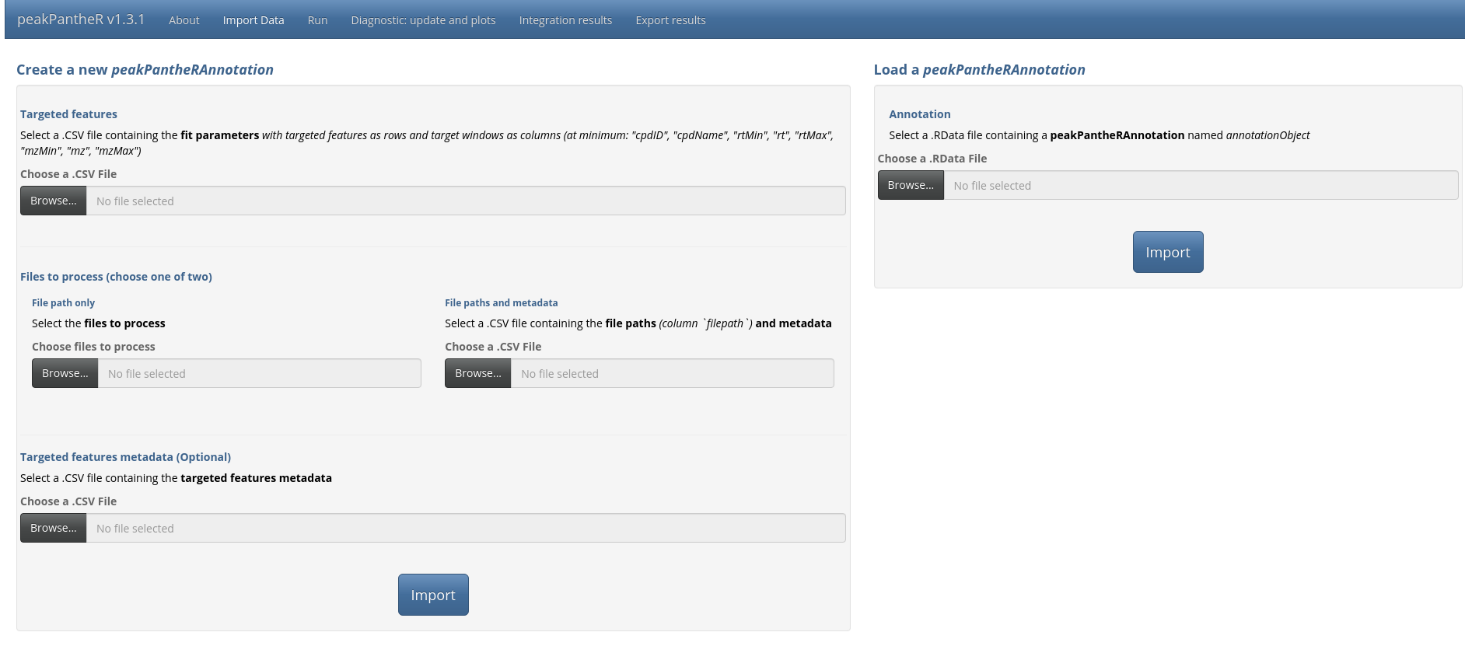


**Figure 12** – The ‘Import Data’ tab, with the .CSV import options (left panel) and .RData import (right panel).

The leftmost panel contains file dialogue boxes for importing a series of .CSV files with the basic information required to initialize a *peakPantheRAnnotation* object. It is divided into three sections, ‘*Targeted Features*’, ‘*Files to process’* and ‘*Targeted features metadata’*. ‘*Targeted features*’ should be a .CSV file containing the ROI information with targeted features as rows and target windows as columns, as shown in ***Table 1***. In the next section, the user can choose to either use a file dialogue button to manually select the files, or import a .CSV. file with a complete list. These options are mutually exclusive. The .CSV file format is recommended for larger datasets, and it allows extra metadata columns to be provided and stored in the *sampleMetadata* attribute of the *peakPantheRAnnotation* object. These columns can be used later in the ‘*Diagnostic: update and plots***’** tab to interactively colour chromatogram plots. Finally, there is an optional file dialogue for extra compound metadata. The metadata will be written in the *cpdMetadata* attribute of the *peakPantheRAnnotation* object and should be provided as a .CSV file with targeted features as rows and metadata fields as columns. The first column should be the *cpdID* as provided in the .CSV file which contains the targeted features ROI information (specified in the ‘*Targeted features’* file dialogue).

The right panel, *‘Load a peakPantheRAnnotation’,* contains a single file dialogue to import a previously initiated *peakPantheRAnnotation* object stored in an .RData file. This can be either a *peakPantheRAnnotation* object generated previously on the command line or via the ShinyGUI.


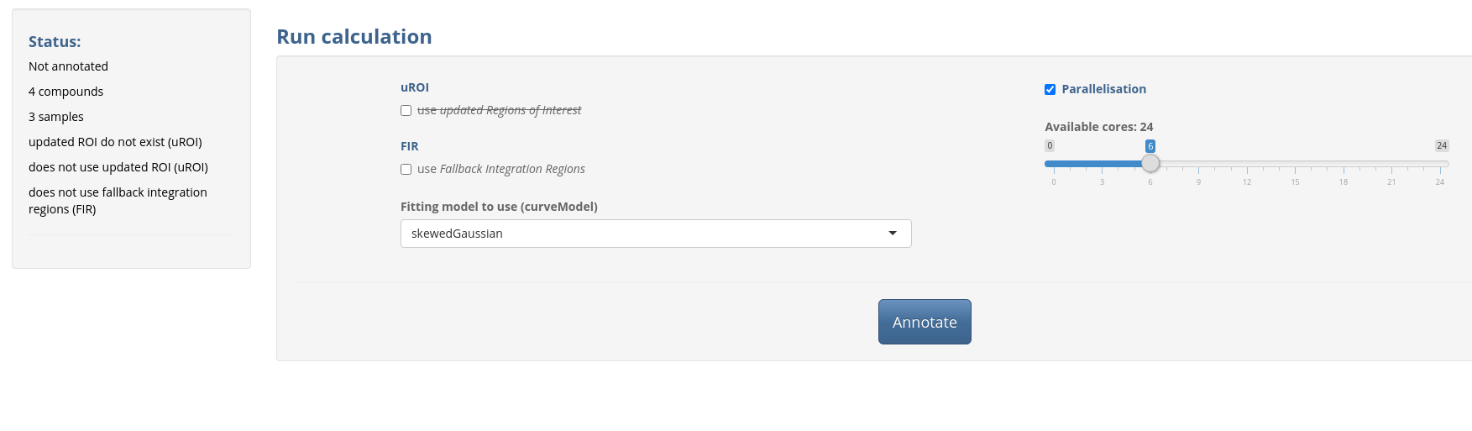


**Figure 13 –** ‘Run’ tab, used to run the peakPantheR annotation workflow.

**Run:**

The *‘Run’* tab (***Figure 9***) is used to execute the *peakPantheR_parallelAnnotation* method, with *n_cores, curveModel, useUROI* and *useFIR* arguments set based on user choice. These parameters are interactively defined n the ‘*Run calculation’* box (***Figure 10***). If the ‘*Parallelisation’* checkbox is ticked, files will be processed in parallel depending on the number of cores selected. The current status of the annotation, including the presence of uROI and FIR information, is displayed on the leftmost ‘*Status’* text box. Both ‘*useUROI*’ and ‘*useFIR’* options should only be toggled if uROI and FIR regions have been defined. Pressing ‘*Annotate’* will trigger the *peakPantheR_parallelAnnotation* method. An animated bar is shown while the annotation process runs in the background.


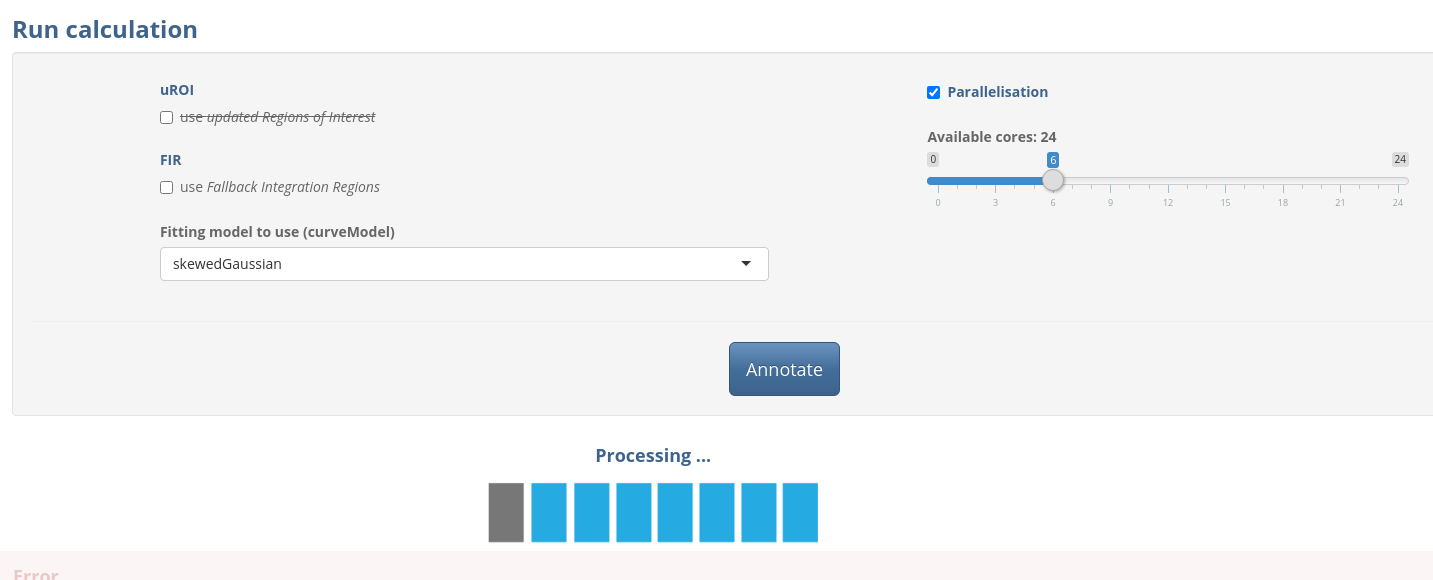


**Figure 14 –** Animated bar shown during the peakPantheRAnnotation_parallelAnnotation method runtime.

**Diagnostic: update and plots:**

This page is divided into 3 tabs, `*Annotation statistics`, `Update uROI/FIR`,* and *`Diagnostic plot`*. The first tab, `*Annotation statistics` (*shown in ***Figure 11***)*,* provides a tabular overview of 4 summary statistics per targeted compound `*CpdID*`, calculated using all integrated samples. `*Ratio peaks found*` specifies the percentage of samples in which it was possible to find a signal in the respective ROI/uROI, while `*Ratio peaks filled*` is the percentage of samples where the curve-fitting procedure failed and fallback integration had to be used instead. *`ppm error`* is the mean deviation, in partsper-million, of the m/z values found from those expected in the uROI/ROI definitions and `*RT deviation (s)`* is the mean difference in seconds between the found “*rt”* values and the “*rt”* as defined in uROI/ROI. These parameters are helpful to flag features for manual review. For example, features with an RT deviation larger than what would be expected based on the chromatographic method’s performance should be investigated to assess if the correct peak is being integrated.


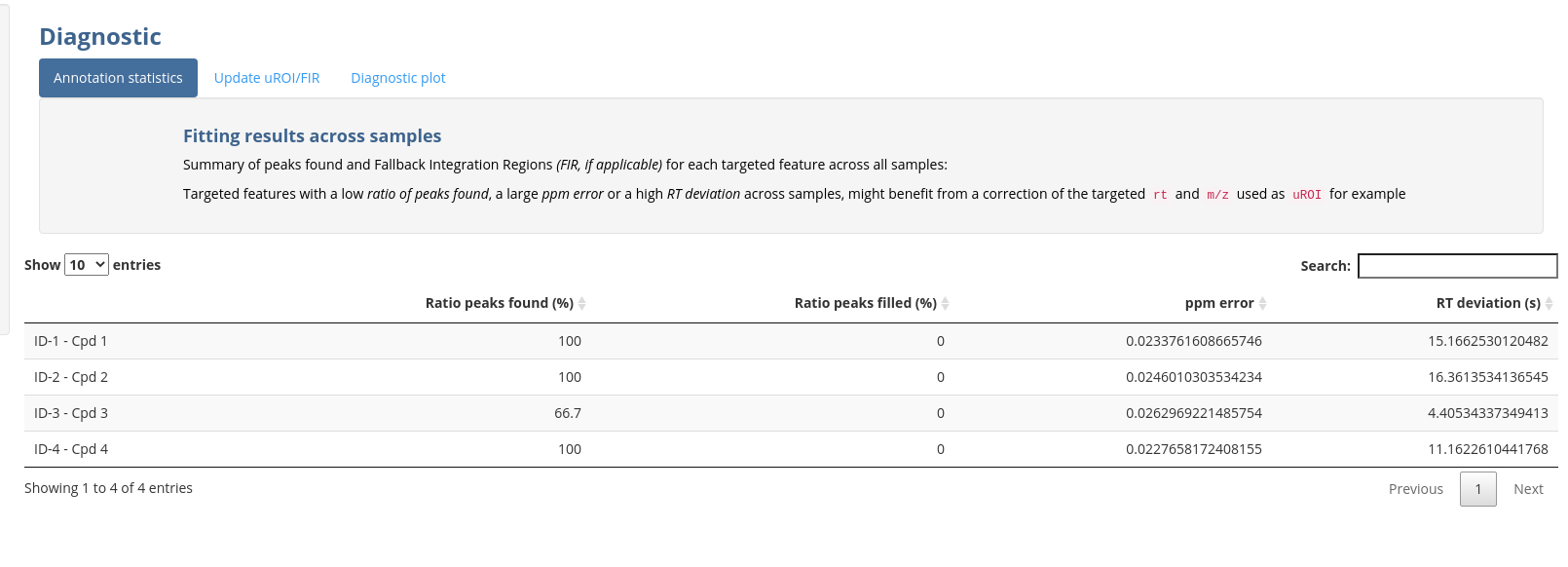


**Figure 15 –** ‘Annotation statistics’ table. Summary statistics calculated from the results of the previous peakPantheRAnnotation are shown for each feature (rows).


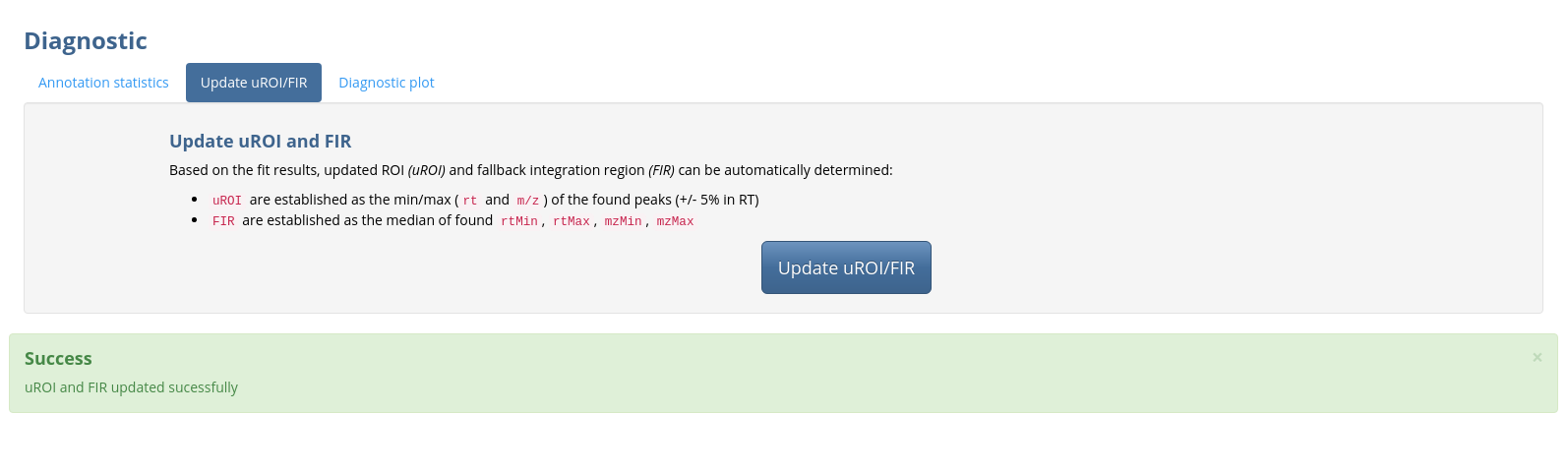


**Figure 16 -** uROI/FIR automatic update. The blue ‘Update uROI/FIR’ button automatically modifies the uROI and FIR information based on the results of a previous run.

The second tab *‘Update uROI/FIR’* (***Figure 12***) contains a single button to automatically update the uROI and FIR settings. This button executes the *annotationParamsDiagnostic,* which automatically modifies the uROI and FIR fields in the *peakPantheRAnnotation* object based on the consensus of a previous run.


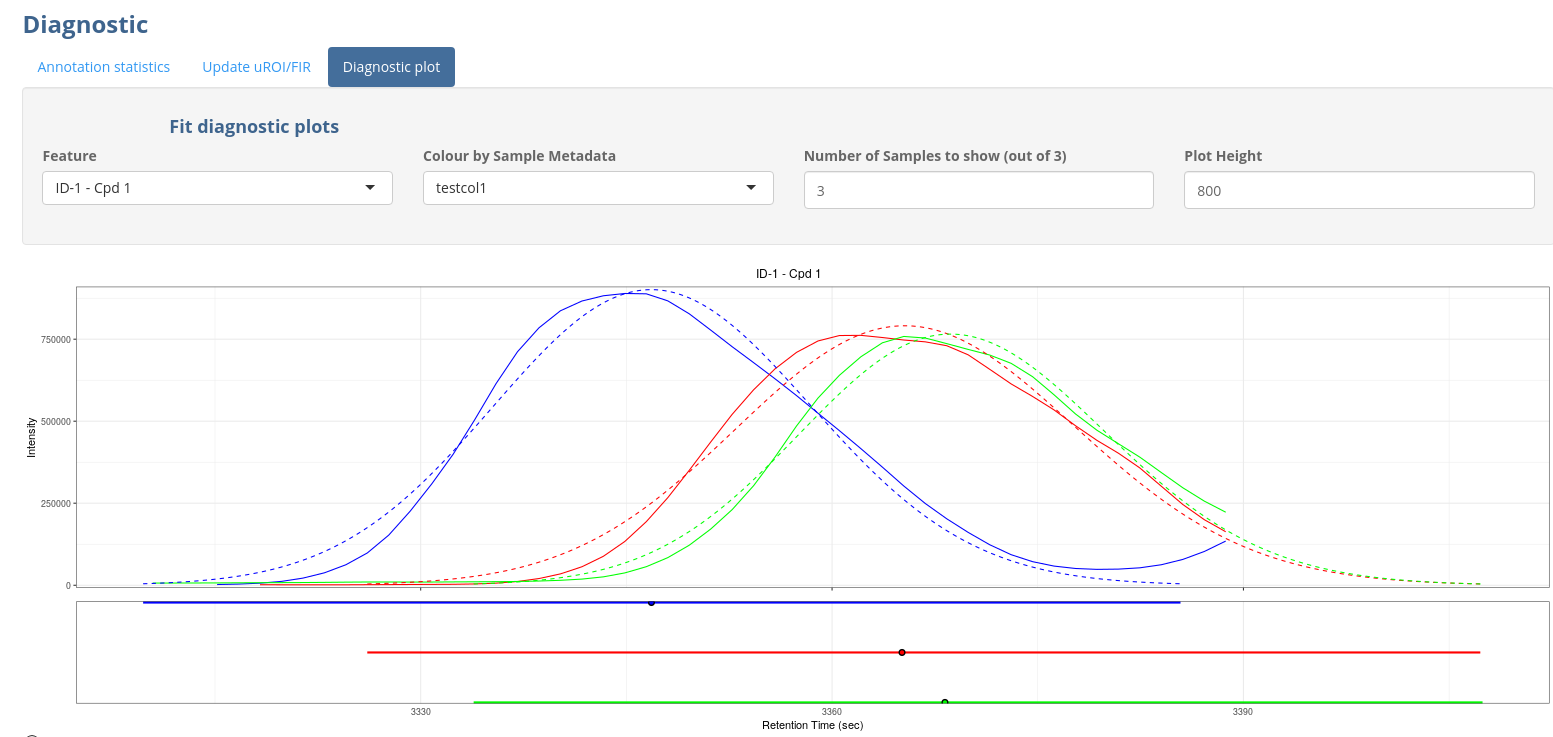


**Figure 17 -** The ‘Diagnostic plot’ tab. In this example, the extracted ion chromatograms for the `ID-1 - Cpd 1` feature from all integrated samples (3 in total) are shown, coloured according to one of the metadata columns, `testcol1`.

Manual refinement of the uROI/FIR windows is sometimes necessary, especially for the retention time boundaries. The *‘Diagnostic plot’* tab (shown in ***Figure 13***) provides visualisation of extracted ion chromatograms and respective fitted peak-shape models, to help the user adjust manually the *rtMin* and *rtMax* parameters. A rectangular box at the top of the page contains a series of input forms and drop-down menus. These include a menu to choose the feature to display (`*Feature`),* a menu to specify if the extracted ion chromatograms should be coloured according to a given metadata field (`*Colour by Sample Metadata*`)*,* and an option to randomly subsample the number of samples plotted, to avoid overcrowding the visualisation plot when assessing results in large datasets (`*Number of Samples to show*`)*.* Additionally, *`Plot Height`* can be used to adjust the aspect ratio of the diagnostic plots.

**Integration results:**

In this page, all the obtained quantities and summary metrics (as shown in ***Table 2*** and ***Table 4***) are displayed in tables. The results can be aggregated across metric, sample, or feature. In the ‘*Overall results’* tab, shown in ***Figure 14***, all observed values for a selected parameter are shown as a data matrix with samples in rows and features in columns. The *‘Results per targeted feature’* (***Figure 15***) and *‘Results per sample’* tabs present the observed values for all metrics (in columns) across either samples or features, respectively (in rows).


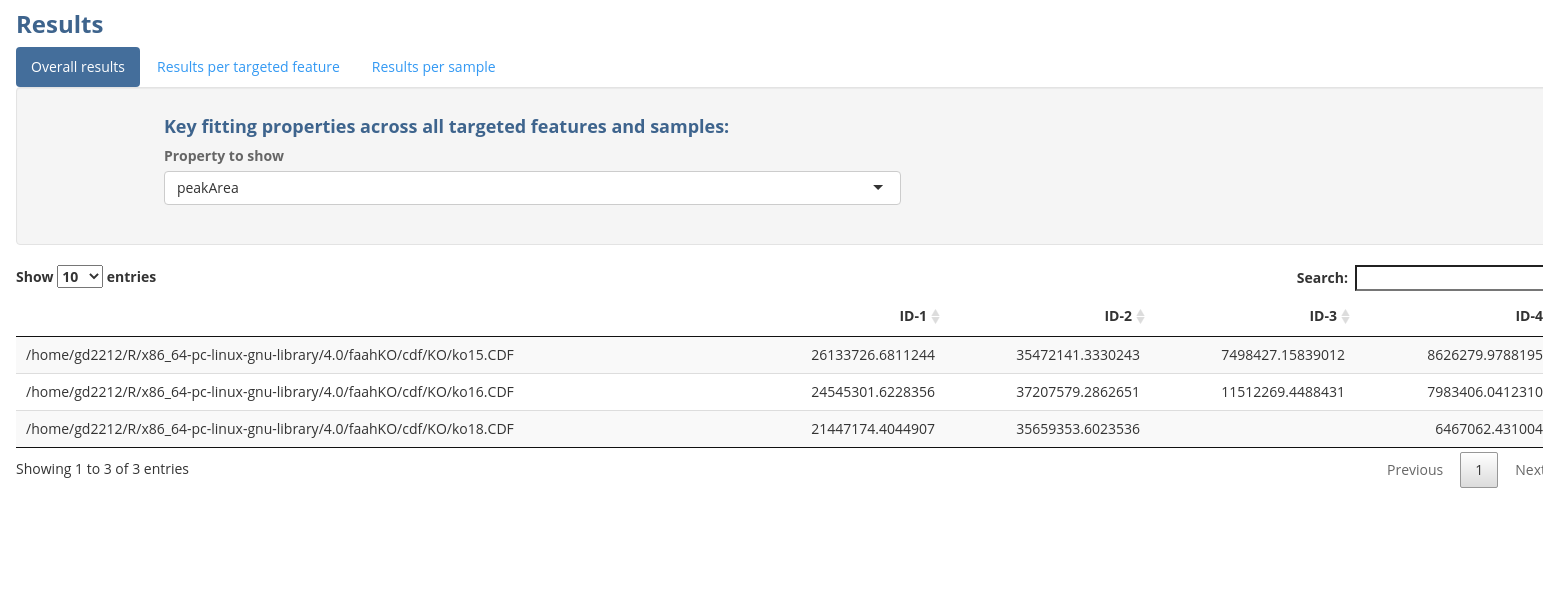


**Figure 18 –** ‘Overall results’ table in the ‘Results’. Samples are shown in rows, and features in columns. The metric to display can be selected via the ‘Property to show’ drop-down menu.


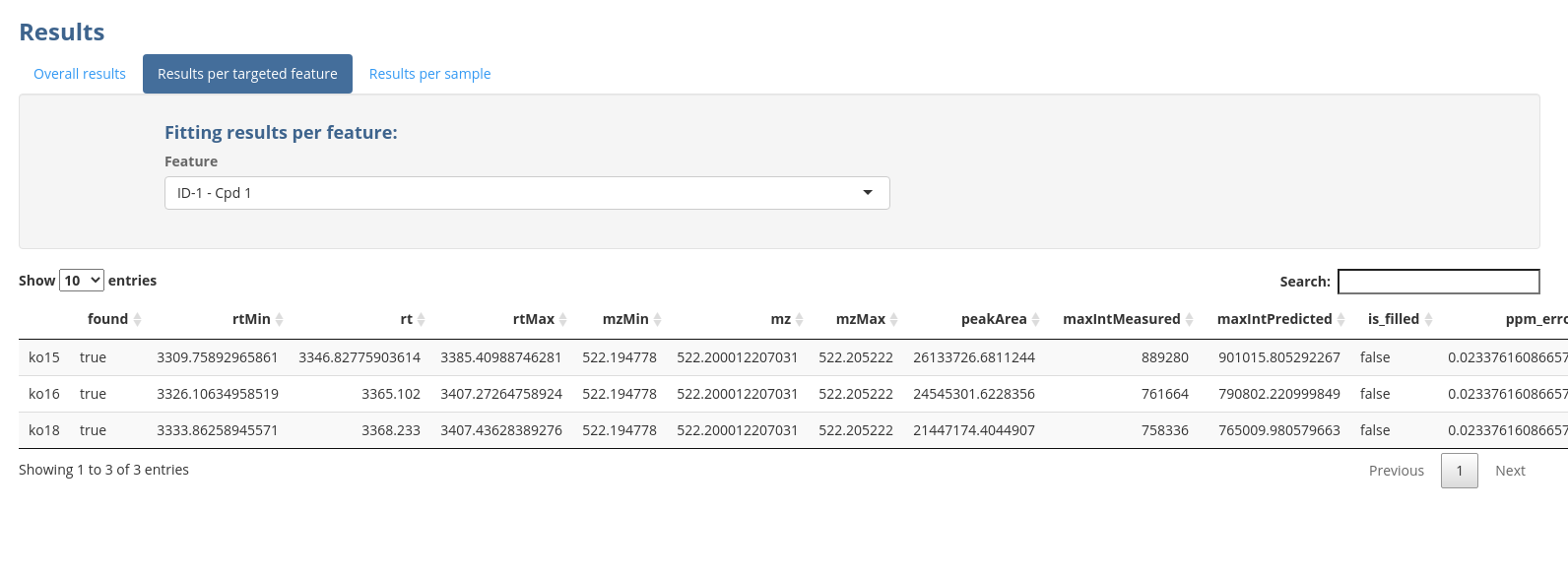


**Figure 19 –** ‘Results per targeted feature’ table, where the values for all metrics (in columns) across samples (rows) are shown for the selected feature.

**Export results:**

Finally, the *‘Export results’* tab, shown in ***Figure 16*** can be used to save all results to .CSV files or .RData files. Under *‘Export Data’,* the ‘*Save annotation as .RData’* panel can be used to export the *peakPantheRAnnotation* object in its current state to an .RData file. This format allows re-opening the object in the peakPantheR GUI in the future and immediately visualising all of the diagnostic plots and annotation results, without having to re-run the workflow. Alternatively, the ROI/uROI/FIR information, the list of files and corresponding *spectraMetadata*, and the *cpdMetadata* can be exported as .CSV files using the blue buttons in the *‘Save input parameters as .CSV’* panel. Compared to the .RData export, the .CSV export has the disadvantage that no information about a previous run is stored, and the diagnostic plots or integration results cannot be inspected without re-running the annotation workflow. Under the ‘*Export Annotation Results and Diagnostic Plots*’ header, the outputs of the o*utputAnnotationDiagnostic* and *exportAnnotationResults* methods mentioned in Section 2 – *“General usage and features”* can be exported through the *‘Save Diagnostic Plots’* and *‘Save Annotation Values’* panels, respectively. The `*Target Folder*’ to save the files is defined in the input form at the bottom of the page.


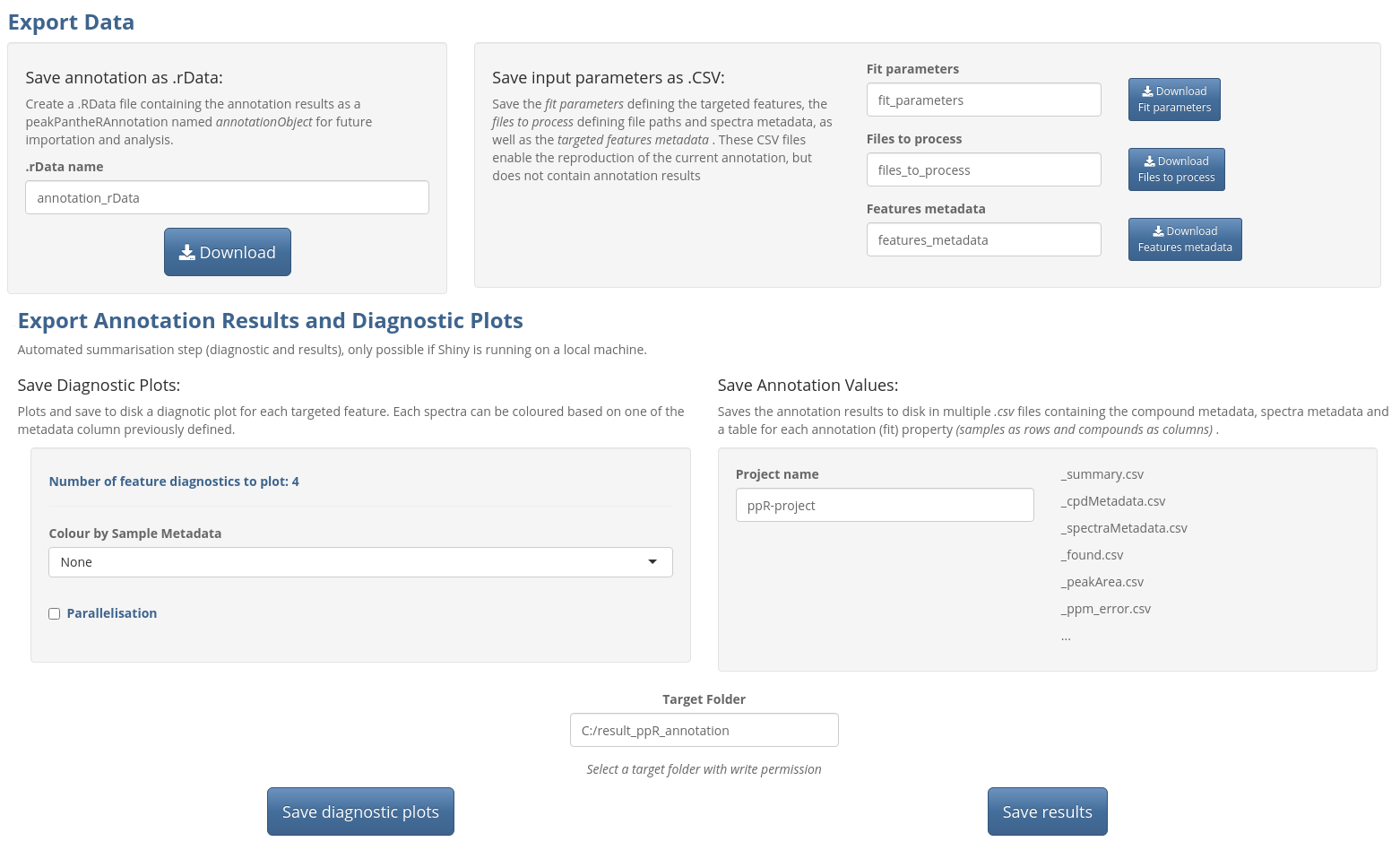


**Figure 20 –** Export results tab. Results from the current run can be exported and re-opened in the future. The diagnostic plots and all data matrices can also be saved to the directory specified on the ‘Target Folder’ entry form, for further inspection or data analysis with other software.

# Experimental details for the dementia cohort dataset and LC-MS metabolite identification

*Chemicals and urine sample analysis*

All mobile phases were prepared with LC-MS grade solvents, formic acid and ammonium formate from Sigma-Aldrich (Dorset, UK). Authentic chemical standards used for the creation of the in-house spectral library (>3000 compounds) were acquired from various sources including, but not limited to, the Mass Spectrometry Metabolite Library (IROA Technologies) and the Aldrich^CPR^ collection. Multiple urine reference materials were developed and used to monitor and evaluate data quality during sample set analysis. Details of the chemical composition and sources of method reference (MR) and internal standards (IS) labelled chemical mixtures are given in Lewis et al^21^ as well as the preparation of quality control Long-Term Reference (LTR) and Study Reference (SR) pooled urine samples.

All urine samples and authentic chemical standards underwent Reversed-Phase (RP) and Hydrophilic Interaction Liquid Chromatography (HILIC) profiling using the methods developed in-house at the National Phenome Centre (NPC) at Imperial College London described in detail in Lewis *et al.* ^21^.

The urine samples set (ALZ) was derived from a UK epidemiological study on dementia. Raw data and more experimental details are available in the MetaboLights Study MTBLS719.

*Metabolite annotation*

Metabolite annotations presented in this work were carried out in the LTR urine sample using a combination of different tools. The list of annotated metabolites along with associated m/z and retention time windows is provided in ***Table S1***.

We used MSI criteria (not revised) to define the level of metabolite annotation^22^. The majority of metabolites were annotated to MSI level 1 using our spectral database and matching retention time (5 s for RP and 15 s tolerance for HILIC profiling, respectively), m/z (15 ppm tolerance for both RP and HILIC methods), MS/MS spectra and isotopic pattern. MSI level 2 annotations consist of metabolites not present in our standards database but for which MS/MS spectra were available in publicly available spectral databases such as HMDB^23^, Metlin^24^, Mass Bank of North America (<https://massbank.us>) and NIST17. The MS/MS spectra for the metabolites annotated at MSI level 3 (three acylcarnitines in HILIC profiling) were searched in published literature and/or predicted using CFM-ID^25^, MAGMa software ^26^, and MetFrag ^27^.

# References

1. R Core Development Team. R: A language and environment for statistical computing. *Vienna, Austria* (2019).

2. Wickham, H. *Advanced R*. *Advanced R* (2014). doi:10.1201/b17487.

3. Chang, W., Cheng, J., Allaire, J. J., Xie, Y. & McPherson, J. shiny: Web Application Framework for R. (2019).

4. Morgan, M. BiocManager: Access the Bioconductor Project Package Repository. (2019).

5. Wickham, H., Hester, J. & Chang, W. devtools: Tools to Make Developing R Packages Easier. (2019).

6. Lovestone, S. *et al.* AddNeuroMed - The european collaboration for the discovery of novel biomarkers for alzheimer’s disease. in *Annals of the New York Academy of Sciences* (2009). doi:10.1111/j.1749-6632.2009.05064.x.

7. Lewis, M. R. *et al.* Development and Application of UPLC-ToF MS for Precision Large Scale Urinary Metabolic Phenotyping. *Anal. Chem.* **88**, acs.analchem.6b01481 (2016).

8. Sands, C. J. *et al.* The nPYc-Toolbox, a Python module for the pre-processing, quality-control and analysis of metabolic profiling datasets. *Bioinformatics* (2019) doi:10.1093/bioinformatics/btz566.

9. Chambers, M. C. *et al.* A cross-platform toolkit for mass spectrometry and proteomics. *Nature Biotechnology* (2012) doi:10.1038/nbt.2377.

10. Gatto, L. & Lilley, K. S. Msnbase-an R/Bioconductor package for isobaric tagged mass spectrometry data visualization, processing and quantitation. *Bioinformatics* (2012) doi:10.1093/bioinformatics/btr645.

11. Elzhov, T. V, Mullen, K. M., Spiess, A.-N. & Bolker, B. minpack.lm: R Interface to the Levenberg-Marquardt Nonlinear Least-Squares Algorithm Found in MINPACK, Plus Support for Bounds. (2016).

12. Wickham, H. ggplot2: elegant graphics for data analysis. (2009).

13. Morgan, M., Obenchain, V., Lang, M., Thompson, R. & Turaga, N. BiocParallel: Bioconductor facilities for parallel evaluation. (2019).

14. Martens, L. *et al.* mzML - A community standard for mass spectrometry data. *Mol. Cell. Proteomics* **10**, R110.000133 (2011).

15. Pedrioli, P. G. A. *et al.* A common open representation of mass spectrometry data and its application to proteomics research. *Nature Biotechnology* (2004) doi:10.1038/nbt1031.

16. Fuhrer, T. & Zamboni, N. High-throughput discovery metabolomics. *Current Opinion in Biotechnology* (2015) doi:10.1016/j.copbio.2014.08.006.

17. Capitanio, A. *The skew-normal and related families*. *The Skew-Normal and Related Families* (2011). doi:10.1017/CBO9781139248891.

18. Abramowitz, M., Stegun, I. A. & Romer, R. H. Handbook of Mathematical Functions with Formulas, Graphs, and Mathematical Tables . *Am. J. Phys.* **56**, 958–958 (1988).

19. More, J. J., Sorensen, D. C., Garbow, B. S. & Hillstrom, K. E. The MINPACK Project. in *Sources and Dev of Math Software* (1984).

20. Fischler, M. a. & Bolles, R. C. Random sample consensus: A Paradigm for Model Fitting with Applications to Image Analysis and Automated Cartography. *Commun. ACM* **24**, 381–395 (1981).

21. Lewis, M. R. *et al.* Development and Application of Ultra-Performance Liquid Chromatography-TOF MS for Precision Large Scale Urinary Metabolic Phenotyping. *Anal. Chem.* **88**, 9004–9013 (2016).

22. Sumner, L. W. *et al.* Proposed minimum reporting standards for chemical analysis: Chemical Analysis Working Group (CAWG) Metabolomics Standards Initiative (MSI). *Metabolomics* **3**, 211–221 (2007).

23. Wishart, D. S. *et al.* HMDB: The human metabolome database. *Nucleic Acids Res.* (2007) doi:10.1093/nar/gkl923.

24. Smith, C. A. *et al.* METLIN: A metabolite mass spectral database. in *Therapeutic Drug Monitoring* vol. 27 747–751 (2005).

25. Allen, F., Pon, A., Wilson, M., Greiner, R. & Wishart, D. CFM-ID: A web server for annotation, spectrum prediction and metabolite identification from tandem mass spectra. *Nucleic Acids Res.* (2014) doi:10.1093/nar/gku436.

26. Verdegem, D., Lambrechts, D., Carmeliet, P. & Ghesquière, B. Improved metabolite identification with MIDAS and MAGMa through MS/MS spectral dataset-driven parameter optimization. *Metabolomics* (2016) doi:10.1007/s11306-016-1036-3.

27. Wolf, S., Schmidt, S., Müller-Hannemann, M. & Neumann, S. In silico fragmentation for computer assisted identification of metabolite mass spectra. *BMC Bioinformatics* (2010) doi:10.1186/1471-2105-11-148.
